# Gene regulation contributes to explain the impact of early life socioeconomic disadvantage on adult inflammatory levels in two cohort studies

**DOI:** 10.1038/s41598-021-82714-2

**Published:** 2021-02-04

**Authors:** Cristian Carmeli, Zoltán Kutalik, Pashupati P. Mishra, Eleonora Porcu, Cyrille Delpierre, Olivier Delaneau, Michelle Kelly-Irving, Murielle Bochud, Nasser A. Dhayat, Belen Ponte, Menno Pruijm, Georg Ehret, Mika Kähönen, Terho Lehtimäki, Olli T. Raitakari, Paolo Vineis, Mika Kivimäki, Marc Chadeau-Hyam, Emmanouil Dermitzakis, Nicolas Vuilleumier, Silvia Stringhini

**Affiliations:** 1 Population Health Laboratory (#PopHealthLab), University of Fribourg, Route des Arsenaux 41, 1700 Fribourg, Switzerland; 2 Center for Primary Care and Public Health (Unisanté), University of Lausanne, Lausanne, Switzerland; 3 Swiss Institute of Bioinformatics, Lausanne, Switzerland; 4 Department of Clinical Chemistry, Fimlab Laboratories, and Finnish Cardiovascular Research Center, Faculty of Medicine and Health Technology, Tampere University, 33520 Tampere, Finland; 5 Center for Integrative Genomics, University of Lausanne, Lausanne, Switzerland; 6 UMR1027, Toulouse III University, Inserm, Toulouse, France; 7 Department of Computational Biology, University of Lausanne, Lausanne, Switzerland; 8 Department of Nephrology and Hypertension, Inselspital, Bern University Hospital, University of Bern, Bern, Switzerland; 9 Service of Nephrology, Geneva University Hospitals, Geneva, Switzerland; 10 Service of Nephrology, Lausanne University Hospital, University of Lausanne, Lausanne, Switzerland; 11 Department of Cardiology, Geneva University Hospital, Geneva, Switzerland; 12 Department of Clinical Physiology, Fimlab Laboratories, and Finnish Cardiovascular Research Center, Faculty of Medicine and Health Technology, Tampere University, 33521 Tampere, Finland; 13 Centre for Population Health Research, University of Turku, Turku University Hospital, Turku, Finland; 14 Research Centre of Applied and Preventive Cardiovascular Medicine, University of Turku, Turku, Finland; 15 Department of Clinical Physiology and Nuclear Medicine, Turku University Hospital, Turku, Finland; 16 MRC Centre for Environment and Health, School of Public Health, Imperial College London, London, W21PG UK; 17 Department of Epidemiology and Public Health, University College London, London, UK; 18 Department of Genetic Medicine and Development, University of Geneva, Geneva, Switzerland; 19 Division of Laboratory Medicine, Geneva University Hospital, Geneva, Switzerland; 20 Unit of Population Epidemiology, Division of Primary Care, Geneva University Hospitals, Geneva, Switzerland

**Keywords:** Computational biology and bioinformatics, Molecular biology, Risk factors

## Abstract

Individuals experiencing socioeconomic disadvantage in childhood have a higher rate of inflammation-related diseases decades later. Little is known about the mechanisms linking early life experiences to the functioning of the immune system in adulthood. To address this, we explore the relationship across social-to-biological layers of early life social exposures on levels of adulthood inflammation and the mediating role of gene regulatory mechanisms, epigenetic and transcriptomic profiling from blood, in 2,329 individuals from two European cohort studies. Consistently across both studies, we find transcriptional activity explains a substantive proportion (78% and 26%) of the estimated effect of early life disadvantaged social exposures on levels of adulthood inflammation. Furthermore, we show that mechanisms other than *cis* DNA methylation may regulate those transcriptional fingerprints. These results further our understanding of social-to-biological transitions by pinpointing the role of gene regulation that cannot fully be explained by differential *cis* DNA methylation.

## Introduction

Inflammatory processes have been suggested as a potential pathophysiological mechanism underlying the development of several major chronic diseases, including cardiovascular, metabolic and psychotic disorders^1^. Systemic inflammation is also one of the most studied pathways in the context of the social-to-biological transition, defined as the ensemble of biological processes and mechanisms involved in the construction of socioeconomic inequalities in health^2–6^. Although early life disadvantaged socioeconomic conditions have been consistently shown to lead to elevated levels of inflammation in adulthood^7^, the underlying biological processes through which those socioeconomic exposures are embodied remain unclear.

Various studies have suggested a dysregulation of gene transcription in leukocytes as a potential biological mechanism for the embodiment of socioeconomic disadvantage^8–10^. This line of reaserch rests on the hypothesis that socioeconomic disadvantage activates the sympathetic nervous system associated to body’s flight or fight response to stressors that eventually leave a leukocyte transcriptional fingerprint. Similar findings have been reported in experiments conducted with macaques^11–13^. Other studies have focused on the epigenetic regulators of transcriptional activity in leukocytes, in particular the DNA methylation levels at specific CpG sites^11,14^. These research endeavors were conducted without directly relating DNA methylation to gene transcription levels, whose functional relationship does not always hold^15,16^.

To date, although discussed theoretically^8^, the contribution of gene regulatory mechanisms underlying socioeconomic inequalities in inflammatory levels has not been investigated empirically, and specifically with data across social-to-biological layers encompassing life course socioeconomic exposures, transcriptomic and epigenetic levels in blood, and circulating inflammatory levels. To explore the chain of biological mechanisms linking early life socioeconomic disadvantage to adult inflammatory levels via DNA methylation and gene transcription in leukocytes, we used data from a Finnish (1,623 participants) and a Swiss (706 participants) population-based cohort study, and evaluated the consistency of findings across these two populations. First, we calculated the portion of the effect of early life socioeconomic disadvantage on adult heightened inflammatory levels explained by transcriptional level of genes either inferred by applying 2-sample Mendelian randomization^17^ on the whole transcriptome (2sMR) or belonging to the conserved transcriptional response to adversity (CTRA) transcriptome profile^18^. We measured levels of systemic inflammation through circulating C-reactive protein (CRP), as it is a sensitive marker of inflammation and elevated amounts have been associated with increased risk for several diseases^1^. Second, we established the most likely functional relationship between *cis* (based on their genomic location) epigenetic and transcriptional changes induced by early life socioeconomic disadvantage via Bayesian networks model selection^19^.

## Results

### *Gene selection *via* 2sMR*

We selected genes based on two strategies independently of the study populations. In one, we defined a set of genes as those having been used in previous research to assess the conserved transcriptional response to adversity (CTRA) transcriptome profile^18^ (see “Materials and methods” section). In the second one, we selected genes based on a transcriptome-wide causal inference analysis via 2-sample Mendelian randomization (2sMR) aiming at establishing leukocytes transcripts (exposure) driving levels of CRP in blood (outcome)^17^. 2sMR is potentially robust with respect to unmeasured confounders and exploits publicly available summary statistics of GWAS. We used summary statistics of the associations between genetic instruments (Single Nucleotide Polymorphisms, SNPs) and gene transcription in leukocytes or CRP in blood obtained via large-scale GWAS conducted by the eQTLGen consortium^20^ and the CHARGE consortium^21^, respectively (see “Materials and methods” section). There were 10,701 genes for which 2sMR provided a test of the causal association between gene transcription and CRP levels. There was an average of 39 semi-independent instruments (*cis* eQTLs in a linkage disequilibrium r^2^ ≤ 0.25, see “Materials and methods” section) per each gene and the average *F*-statistic was 97. Finally, 81 genes were significant at *q* ≤ 0.05 for the three applied MR methods, namely random effect inverse variance weighting, Egger regression, and penalized weighted median regression (see “Materials and methods” section for further details and Table S1). Of note, one of them, *NFKB1*, was as well one of the CTRA indicator genes. Functional enrichment analysis revealed enriched inflammatory bowel disease (adjusted *P* value = 0.016), NLRP3 inflammasome (adjusted *P* value = 0.045), ABIN2-NFKB1-MAP3K8 complex (adjusted P value = 0.0498), and prion disease pathways (adjusted P value = 0.0499).

### Characteristics of populations

As reported in Table 1, on average Finnish participants (YFS study) were 42-year old (age ranging between 34 and 49 years), while Swiss participants (SKIPOGH study) were 53-year old (age ranging between 25 and 88 years). Women represented 46% of the YFS population and 52% of the SKIPOGH population. CRP levels in SKIPOGH were distributed differently than in YFS, as participants in the upper quintile had CRP ≥ 3 mg/L in SKIPOGH while had CRP > 2.2 mg/L in YFS (see Table 1 and Supplementary Information for a discussion about this inter-cohort difference). Individuals with high/low parental occupational position were 18%/26% in YFS and 25%/28% in SKIPOGH.

**Table 1:** Characteristics of the YFS and SKIPOGH populations.

Characteristics	YFS	SKIPOGH
N	1623	706
Parental occupational position [high/intermediate/low]	289 (17.8%)	176 (24.9%)
	907 (55.9%)	329 (46.6%)
	427 (26.3%)	201 (28.5%)
Parental education [high/intermediate/low]	439 (30.9%)	187 (26.5%)
	480 (33.7%)	216 (30.6%)
	504 (35.4%)	303 (42.9%)
Center	238 (14.7%)	106 (15.0%)
	343 (21.1%)	290 (41.1%)
	278 (17.1%)	310 (43.9%)
	503 (31.0%)	
	261 (16.1%)	
Sex [man/woman]	882 (54.3%)	341 (48.3%)
	741 (45.7%)	365 (51.7%)
Age [years]	42 [34,49]	53 [25,88]
CRP [mg/L]	0.7 [0.3,2.2]	CRP < 3 mg/L: 560 (79.3%)
		CRP ≥ 3 mg/L: 146 (20.7%)

Participants are those with complete information about parental occupational position, transcriptome and CRP. Categorical characteristics are summarized through their absolute and relative (%) prevalence. Age is summarized through its mean and range. CRP in YFS is summarized with median and lower to upper quintiles range, while in SKIPOGH is binarized as 3 mg/L is the highest left truncation value (see “Materials and methods” section).

### Mediation by gene transcription

**Figure 1:**
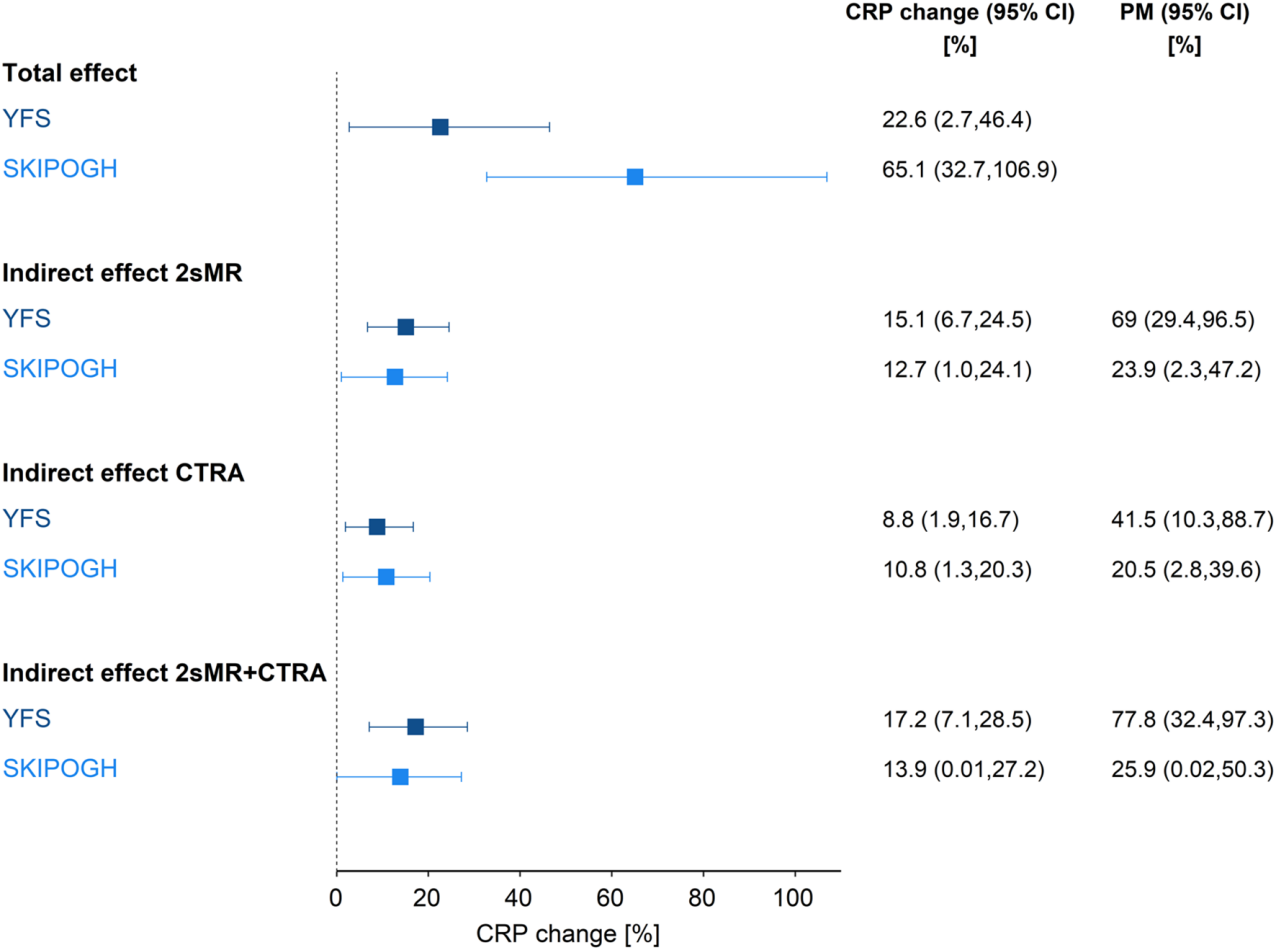
Amount of mediation by gene regulation. Size of effects and proportion mediated (PM) estimated by mediation analysis in each population. CRP change (%) and 95% confidence intervals (CI) are reported for the total effect of low (vs high) parental occupational position on CRP levels in adulthood, and for indirect effects through transcription levels of selected genes *en-bloc*. Joint indirect effects via 2sMR, CTRA or 2sMR + CTRA genes were estimated by summarizing transcription levels with principal components corresponding to about 50% of the transcription variance in each genes’ set.

Individuals with low parental occupational position had an increase in (the geometric mean of) adulthood CRP of 22.6% [95% confidence intervals (CI): 2.7%, 46.4%] for YFS and 65.1% [95% CI: 32.7%,106.9%] for SKIPOGH compared to having high parental occupational position (total effect, see Fig. 1). The point estimate of the geometric mean of CRP for individuals with low parental occupational position was 0.75 mg/L in YFS and 0.52 mg/L in SKIPOGH, while for individuals with high parental occupational position was 0.61 mg/L in YFS and 0.32 mg/L in SKIPOGH.

The portion of the total effect accounted by 2sMR-implicated genes *en-bloc* (indirect effect, whereby transcription levels of 2sMR genes were summarized by the first 23 principal components (PCs) corresponding to 49.9% of transcription variance, see “Materials and methods” section) resulted in an increased (geometric mean of) CRP of 15.1% [95% CI: 6.7%, 24.5%] in YFS, and of 12.7% [95% CI: 1.0%,24.1%] in SKIPOGH (first 23 PCs corresponding to 50.4% of transcription variance). This corresponded to a proportion mediated (PM, see “Materials and methods” section) of 69.0% [95% CI: 29.4%, 96.5%] in YFS and of 23.9% [95% CI: 2.3%, 47.2%] in SKIPOGH. The indirect effect accounted by CTRA indicator genes *en-bloc* (first 12 PCs corresponding to 49.3% of transcription variance) resulted in an increased (geometric mean of) CRP of 8.8% [95% CI: 1.9%, 16.7%] in YFS, and of 10.8% [95% CI: 1.3%, 20.2%] in SKIPOGH (first 10 PCs corresponding to 49.8% of transcription variance). The corresponding PM was 41.5% [95% CI: 10.3%, 88.7%] in YFS and 20.5% [95% CI: 2.8%, 39.6%] in SKIPOGH. When considering 2sMR-implicated and CTRA indicator genes together (133 in total, see Table S1), their joint indirect effect resulted in an increased (geometric mean of) CRP of 17.2% [95% CI: 7.1%, 28.5%] in YFS (first 33 PCs corresponding to 50.1% of transcription variance) and of 13.9% [95% CI: 0.0%, 27.2%] in SKIPOGH (first 30 PCs corresponding to 49.8% of transcription variance). This corresponded to a PM of 77.8% [95% CI: 32.4%, 97.3%] in YFS and of 25.9% [95% CI: 0.02%, 50.3%] in SKIPOGH. The partial redundancy between the two gene sets was supported by correlation patterns between the principal components and between the transcription levels (see Figures S1 and S2).

Overall, in both cohorts the magnitude of effects was in the same direction and that of indirect effects was similar, with estimates of 17.2% and 13.9% indicating that gene transcription in leukocytes mediates a substantive portion of the estimated total effect of parental occupational position on adult CRP.

### Regulation of gene transcription by local DNA methylation

The analysis of the relationship between parental occupation position and transcription levels of the 2sMR-implicated and CTRA indicator genes revealed a small group of genes significantly associated at *P* value ≤ 0.05. In YFS, there were twenty-three genes, among which seventeen/six had decreased/increased transcription levels for low vs high parental occupational position. In SKIPOGH, there were thirteen genes, among which four/nine had decreased/increased transcription levels for low vs high parental occupational position. The genes common across both populations were *DDR1, IFI27,* and *IFIT1L.*

To explore the role of DNA methylation linking early life socioeconomic disadvantage to transcription levels in adulthood, we applied Bayesian network scoring to 4,076 CpGs located nearby (< 1500 bp) the 2sMR-implicated and CTRA indicator genes (see Table S2), for five causal structures (Group A to E, see Figure S5 and “Materials and methods” section). As described in Table 2, for most CpGs (Group A: N = 3612 (88.6%) and N = 3253 (79.8%) in YFS and SKIPOGH, respectively), there was no substantive evidence (logarithm of Bayes factor larger than one, see “Materials and methods” section) for an influence of parental occupational position on adult regulation of gene transcription. For one CpG (cg19527571), there was evidence for the role of DNA methylation as *cis*-regulator of *MED24* in SKIPOGH only (Group B). There was evidence for parental occupational position driving levels of gene’s transcription and DNA methylation being either unrelated to transcription (Group C: N = 30 and N = 36 CpGs in YFS and SKIPOGH, respectively) or driven by transcription (Group D: N = 11 and N = 17 CpGs in YFS and SKIPOGH, respectively). For 9 and 24 CpGs (Group E, YFS and SKIPOGH, respectively) there was evidence for parental occupational position driving levels of methylation but not transcription levels. Finally, there was no substantive evidence for one causal structure over the others in N = 414 (10.2%) and N = 745 (18.3%) CpGs in YFS and SKIPOGH, respectively.

**Table 2:** Cardinality of the most likely group of model structures across the five tested competing groups (see Figure S5) for a total of 4076 CpG sites in up to N = 1367 YFS and N = 661 SKIPOGH participants.

Causal structures		No. of CpGs	
		YFS	SKIPOGH
A	Parental occupational position does not influence methylation nor transcription levels	3,612	3,253
B	Parental occupational position drives changes in methylation and transcription levels; methylation also causes changes in transcription	0	1
C	Parental occupational position drives gene transcription, but methylation and transcription are not related	30	36
D	Parental occupational position drives transcription that in turn influences methylation levels	11	17
E	Parental occupational position influences methylation but not transcription levels	9	24
None		414	745
Total		4,076	4,076

The most likely group of causal structures is selected when its posterior probability is at least roughly three times larger than any other group’s posterior probability. None corresponds to CpGs for which none of the five tested groups reached winning evidence or models could not be estimated as the transcription level was not available in a few genes (37 and 33 CpGs in YFS and SKIPOGH, respectively).

Overall, a non-empty set of CpGs was present across the two cohort studies for Groups A, C, D and E. Furthermore, this consistency at pattern level did correspond to a replication at the level of single site for 2,894 CpGs belonging to Group A only (see Table S2).

Compared to CpGs in Group A, sites in either Group C or D (N = 41 and N = 53 in CRYFS and SKIPOGH, respectively) had similar distribution in CpG island position (island or shore or shelf vs open sea, χ^2^ = 0.1 and 0.2, *P* value = 0.76 and 0.48, %, YFS and SKIPOGH, respectively) and increased TSS or UTR annotation (56% vs 44% and 64% vs 44%, χ^2^ = 2.8 and 7.9, *P* value = 0.15 and 0.005, YFS and SKIPOGH, respectively).

### Sensitivity analyses

Total and indirect effect estimates were in the same direction and of similar (for YFS) and higher (for SKIPOGH) magnitude to those reported in main analysis when excluding participants with high values of CRP (> 10 mg/L, N = 34 [2.1%] in YFS and N = 12 [1.7%] in SKIPOGH) (see Figure S3). This finding supports the fact that our main results do not reflect short term immune responses.

By varying the amount of variance explained by principal components summarizing transcription levels of the genes’ sets, indirect effect estimates slightly increased when variance moved from 30 to 50% and remained stable when variance moved from 50 to 60% (see Table S3). This finding supports that indirect effects reported in main analyses do not underestimate the amount of mediation by gene regulation in leukocytes. In SKIPOGH, when using a dichotomous CRP (≥ 3 mg/L) as outcome, the estimated indirect effect was substantive (see Table S4), backing the results presented in main analyses with an imputed continuous CRP.

When running a sequential mediation analysis to assess the effect not mediated by adulthood occupational position, the indirect effect through adulthood occupational position was 7.5% in YFS and 3.9% in SKIPOGH, while it increased to 26.5% in YFS and 15.5% in SKIPOGH when both adulthood occupational position and transcription levels of genes in leukocytes were included as the mediators (see Table S5). This substantive additional mediation supported the presence of a mediation by transcription levels of genes in leukocytes independent of adulthood occupational position, or alternatively that only a small fraction of the effect of parental occupational position on CRP was explained by adulthood occupational position.

When running a sequential mediation analysis to assess the path-specific effect not mediated by leukocyte composition in blood, the indirect effect through leukocyte composition in blood was − 2.4% in YFS and -0.8% in SKIPOGH, while it increased to 18.0% in YFS and 11.6% in SKIPOGH when both leukocyte composition in blood and transcription levels of genes in leukocytes were the mediators (see Table S6). This substantive additional mediation supported the presence of a pathway going from paternal occupational position to CRP via transcription levels of genes in leukocytes not passing through leukocyte composition in blood, or alternatively that transcription levels of genes in leukocytes and not leukocyte composition in blood truly mediates the effect of parental occupational position on CRP*.*

When parental education was considered as the exposure, the magnitude of indirect effects was in the same direction and slightly lower than that reported in main analysis (see Figure S4). Furthermore, the Bayesian scoring procedure supported a pattern of models similar to the one found for parental occupational position, but showed no consistent evidence for models whereby parental education drives transcription which then affects DNA methylation levels (see Table S7). Overall, these findings support the fact that our main results are consistent across different proxies for early life socioeconomic conditions.

When adding a group of negative control DAGs to the groups described in Figure S5, the Bayesian scoring procedure correctly did not assign that group to any of the CpGs. This finding supports the ability of the applied Bayesian network model selection procedure to identify plausible dependency structures from the data. When adding leukocyte composition as a potential confounder of the relationship between methylation and transcription levels, the Bayesian scoring procedure supported a pattern of models similar to the one reported in main analysis (see Table S8). This finding supports our main result of the role of DNA methylation in regulating nearby gene transcription and is robust despite the potential confounding of leukocyte composition in blood. Finally, when selecting *cis* CpGs within 5,000 bp of the annotated gene location, the Bayesian scoring procedure supported a pattern of models similar to the one reported in main analysis (see Table S9), suggesting that our findings are not sensitive to the choice of a threshold to select *cis* CpGs.

## Discussion

We found that both Finnish and Swiss adults having experienced disadvantaged socioeconomic conditions early in life displayed heightened levels of systemic inflammation (CRP) compared to their more advantaged counterparts, and that a substantive portion of this effect was transmitted by shifts in leukocytes’ gene transcription. Via 2sMR, we identified a new set of CRP-related genes putatively influenced by socioeconomic conditions in early life. Finally, we showed a consistent pattern of inter-individual variation in *cis* DNA methylation being either unrelated to or driven by inter-individual variation in transcription.

To our knowledge, this is the first study examining gene regulatory mechanisms in leukocytes linking early life socioeconomic conditions to inflammatory levels in adulthood by integrating life course socioeconomic exposures, epigenetic and transcriptomic profiling from blood, and circulating inflammatory levels in two European populations. The observed socioeconomic inequalities in CRP (that is the estimated total effect) were in line with a previous multi-cohort study (N = 13,078) from four European countries^22^, whereby the pooled association between paternal occupational position and CRP resulted in an increased (geometric mean of) CRP of 20.9% [95% CI: 11.6%, 31.0%]. In our study, some differences across the two cohorts were observed in the estimated magnitude of total and indirect effects. As populations were sampled using different designs, were from different European countries and had a different age distribution, these characteristics may have influenced those inter-cohort differences. Remarkably, in both populations those effects were in the same direction and the magnitudes of mediation were similar. Indeed, the joint indirect effect estimates were 17.2% and 13.9% (2sMR + CTRA genes in YFS and SKIPOGH, respectively), indicating a substantive mediation. We acknowledge that the magnitude of the indirect effects may be an overestimate since unmeasured confounding may have increased the association between gene transcription and heightened inflammation. However, at the same time the magnitude could have been underestimated as we captured 50% of the transcription variance via principal components. Based on a sensitivity analysis, the latter case seems to be unlikely. Furthermore, another limitation of our study was that CRP was not measured through a high sensitivity assay in SKIPOGH. However, main and sensitivity analysis provided consistent results, and two thirds of SKIPOGH participants had CRP measured with high precision at 2 mg/L (coefficient of variation < 5%). Additionally, the two European populations were composed of only White participants, which could limit the generalisability of our results to non-White populations. Lastly, the ability to investigate a greater number of genes/genes sets in mediation models (e.g. sets of thousands of genes related to the immune response) was limited, given modelling constraints with a large number of mediators and the available sample size^23^. Future studies should consider a broader range of gene sets. However, overall our finding of a mediating role of gene regulation in leukocytes is supported by previous studies in humans and primates. Those research endeavours pinpointed transcriptional fingerprint of decreased glucocorticoid and increased pro-inflammatory signalling as key mechanism in the embodiment of socioeconomic disadvantage^8,11^.

Our transcriptome-wide analysis also identified 81 CRP-related genes via 2sMR, a method that is protected from confounding and reverse causation biases. By using summary statistics from two large data sets we increased statistical power, and by performing inference via three 2sMR methods based on orthogonal assumptions we strengthened the reliability of inference. Nevertheless, we cannot exclude bias in our 2sMR analysis due to selection of instruments in the same data set where we conducted inference^17^, which leads to underestimation of causal effect sizes. Furthermore, we applied a stringent, rather conservative threshold for selecting genes, and we could not apply 2sMR to all human genes, therefore we cannot exclude the existence of other undetected CRP-related genes.

We showed in both Finnish and Swiss individuals that early in life socioeconomic conditions may regulate gene transcription in leukocytes without involving *cis* DNA methylation changes, or with *cis* DNA methylation playing the role of regulated event. Unmeasured confounding may have biased our results, as genetic variation associated to both DNA methylation and gene transcription levels. However, our finding is in line with recent observations obtained via a novel sequencing technology^15^, Bayesian network analyses of ancestry-related differences in DNA methylation/gene transcription in cord blood^24^ and whole blood monocytes^25^, and a Mendelian randomization study^26^. As pointed out therein^26^, the inferred direction of causality between DNA methylation and gene transcription can be biased because their measurement is noisy. Nevertheless, our findings are based on interpreting only models with substantial statistical evidence (logarithm of Bayesian factor larger than one). At the same time, that threshold could have biased our findings, as we could not assign any of the investigated causal structures to about 10% of the CpGs. Our results do not exclude the possibility that at some *cis* CpGs methylation levels do drive gene transcription and they point to the presence of other regulating mechanisms. Overall, these findings call attention to potential weaknesses of current epidemiologic approaches based on investigating a single epigenetic layer uncoupled with downstream functional molecular phenotypes.

Our study supports regulation of genes in leukocytes as a mechanism through which disadvantaged early life socioeconomic conditions heighten inflammatory levels in adult life, but it is still unclear when that happens and via which intermediate exposure. Indeed, in our analyses occupational position in adulthood mediated only a small portion of the effect of parental occupational position on CRP. The investigation of other factors related to the adult environment and repeated measurements across the life course are needed to answer that question. Our work could extend in prioritizing repeated measurements in life course longitudinal studies to identify critical/sensitive windows of socioeconomic exposures.

## Materials and methods

### Study populations

We used individual-participant data from two multi-centre population-based studies: the Cardiovascular Risk in Young Finns Study (YFS)^27^ and the Swiss Kidney Project on Genes in Hypertension (SKIPOGH)^28^.

YFS participants were selected from five Finnish areas according to the location of the university cities with a medical school (Helsinki, Kuopio, Oulu, Tampere and Turku). In each area, urban and rural boys and girls were randomly selected on the basis of their personal social security number from the Social Insurance Institution's population register, which covers the whole Finnish population. The baseline survey was conducted in six age cohorts of children (aged 3, 6 and 9) and adolescents (aged 12, 15 and 18) in 1980, with a participation rate of 83%, i.e. 3,596 of those invited. Participants were re-examined every 3 years between 1980 and 1992, in 2001 and in 2011, when they had reached mid adulthood (aged 34–49). The YFS study was conducted according to the guidelines of the Declaration of Helsinki, and the protocol was approved by ethics committees of University of Helsinki, Kuopio, Oulu, Tampere and Turku. Informed consent was obtained for all participants and from parents when subjects were under 18.

SKIPOGH participants were recruited in the cantons of Bern and Geneva and the city of Lausanne, Switzerland. Participants were randomly selected from the population-based CoLaus study in Lausanne^29^, and from the population-based Bus Santé study in Geneva^30^. In Bern, participants were randomly selected using the cantonal phone directory. Inclusion criteria were: (i) written informed consent; (ii) minimum age of 18 years; (iii) white ethnicity; (iv) at least one, and preferably three, first-degree family members also willing to participate. Participation rate was 20% in Lausanne, 22% in Geneva, and 21% in Bern. The baseline assessment initiated in December 2009 and ended in April 2013, including 1,128 participants aged between 18 and 90 years. A follow-up survey (1,033 individuals) started in October 2012 and was completed in December 2016. The SKIPOGH study was approved by the ethical committees of Lausanne University Hospital, Geneva University Hospitals and Bern University Hospital. Informed consent was obtained for all participants and research was performed in accordance with relevant guidelines and regulations.

### Causal models and measures

**Figure 2:**
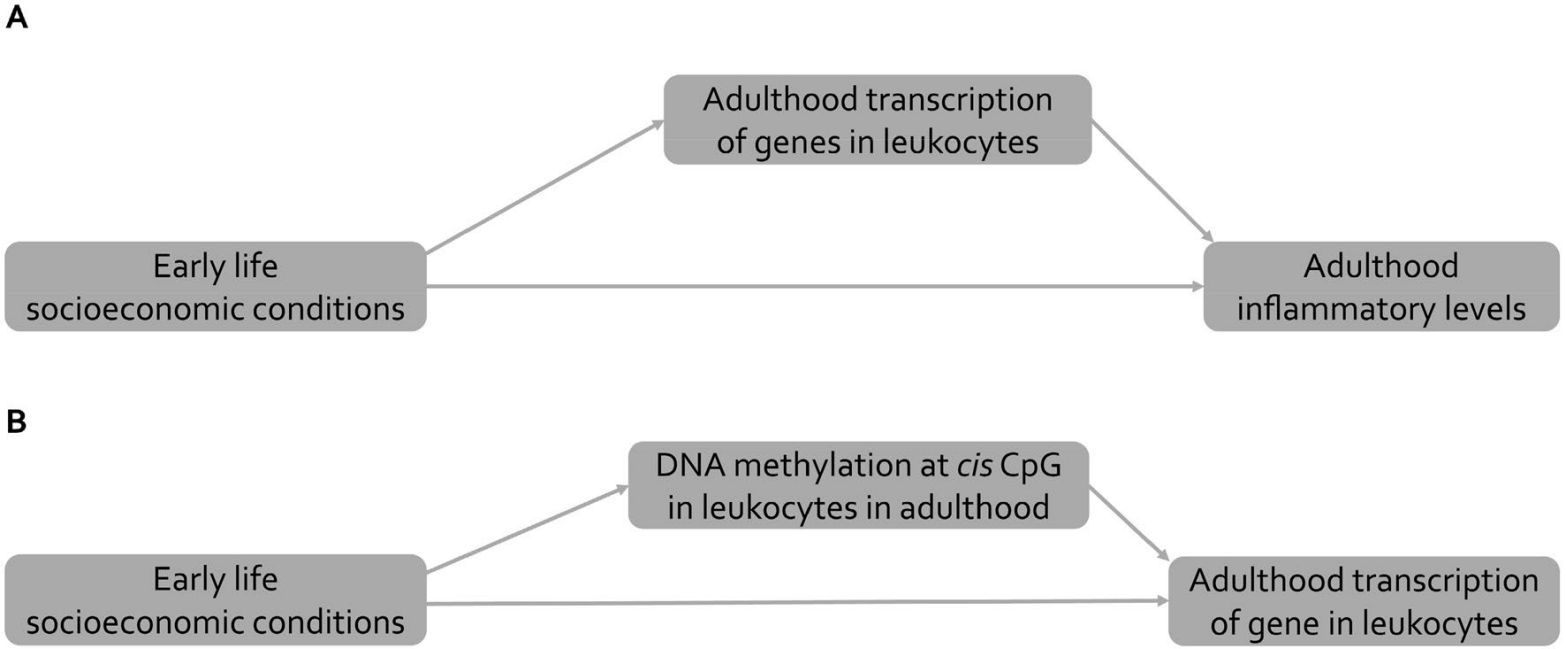
Causal structures. For the sake of simplicity, we do not draw confounders (age, sex, centre of blood collection). Early life socioeconomic conditions represent the exposure. (**A**) Inflammatory levels in adulthood represents the outcome, and adulthood transcription of genes in leukocytes the investigated intermediate mechanism. (**B**) Transcription level of a gene in leukocytes represents the outcome and DNA methylation levels at a specific *cis* CpG in leukocytes the mediating mechanism.

The causal structures posited in our study are represented in the directed acyclic graphs (DAGs) displayed in Fig. 2. The DAG in Fig. 2A evaluates the portion of the effect of early life socioeconomic conditions (exposure) on adult inflammatory levels (outcome) transmitted by intracellular processes regulating transcription of genes in immune cells (mediators). This “indirect” effect captures all potential pre- and post-transcriptional regulating mechanisms including DNA methylation, histone modifications, transcription factors binding and microRNAs^18^. These processes mutually regulate in both physiological and diseases conditions. In the second part of our study, we focused on local DNA methylation as regulating mechanism (mediator) of a gene’ transcription (outcome) according to the causal structure in Fig. 2B. We chose DNA methylation as it is the most widely studied epigenetic mechanism in the field of social epidemiology and as it represents a potential therapeutic target^31^. Since the relationship between levels of local DNA methylation and gene transcription can be bidirectional^24,25^ or absent^15^, for each gene and DNA methylation site we did not posit any specific causal structure between gene transcrition and DNA methylation measurements. Instead, we investigated the most likely one among all possible causal structures (see Figure S5 and section entitled *Bayesian network model selection*).

Parental occupational position defined as the highest household occupation was used as an indicator of socioeconomic conditions in early life (main analysis). In YFS, the occupational position (manual, lower-grade non-manual and higher-grade non-manual, farmer) of the head of the household was assessed in 1980 (study’s baseline), while SKIPOGH participants reported at the study’s follow-up the profession of their father when they were children. Paternal occupational position is a commonly used indicator of socioeconomic conditions in early life^32^ and is closely related to parental occupational position in the Swiss context until the early 1980s (see Supplementary Information). In a sensitivity analysis, we adopted a measure of parental education as an alternative proxy of socioeconomic conditions in early life (see SupplSupplementary Information).

Occupational position was categorized according to the European SocioEconomic Classification (ESEC) scheme into high (higher professionals and managers, higher clerical, services, and sales workers (European socioeconomic class 1–3)), intermediate (small employers and self-employed, farmers, lower supervisors and technicians, class 4–6), or low (lower clerical, services, and sales workers, skilled workers, semi-skilled and unskilled workers, class 7–9)^33^.

Systemic inflammatory status was assessed through the amount of C-reactive protein (CRP [mg/L]) in serum (YFS) and plasma (SKIPOGH). CRP is a sensitive marker of inflammation and elevated amounts of CRP have been associated with increased risk for several diseases^1^. In YFS, CRP levels were measured from blood collected in 2011 through an automated analyzer (Olympus AU400; Tokyo, Japan) and a highly sensitive turbidimetric immunoassay kit (‘CRP‐UL’‐assay; Wako Chemicals, Neuss, Germany). Detection limit of the assay was 0.06 mg/L.

In SKIPOGH, CRP levels were measured from blood collected between 2012 and 2016 through standard immunoturbidimetry with different detection limits (1 mg/L or 2 mg/L or 3 mg/L) depending on the centre or diagnostic machine used (Cobas 8000 Roche Diagnostics, Modular Roche Diagnostics, Beckmann). Inter-assay coefficients of variation were 7.5% at 10 mg/L for N = 317 (Beckmann), 3.3% at 1.5 mg/L for N = 372 (Modular Roche), and 4.0% at 0.9 mg/L for N = 344 (Cobas 8000 Roche). Since ~ 60% of SKIPOGH participants had CRP values below the detection limits, we imputed left censored values via censored maximum likelihood multiple imputation^34^. We ran 30 imputed data sets with a model of CRP including paternal occupational position (or parental education), body mass index, age, sex, and centre as predictors. Parameters (effects described in section *Mediation approach*) were estimated for each data set of imputed CRPs and pooled following Rubin’s rule.

Gene transcription and DNA methylation in adulthood were considered as potential mediators and were measured from a mixture of leukocytes cells collected in 2011 for YFS and between 2012 and 2016 for SKIPOGH. Transcriptomic data were available for 1,650 YFS and 723 SKIPOGH participants via Illumina chip and sequencing technologies, respectively (see Supplementary Information). DNA methylation was measured in 1,548 YFS and 701 SKIPOGH participants via Illumina Infinium chips from the same blood samples transcriptomic data were generated (see Supplementary Information).

Age, sex, centre of blood collection were included as potential confounders.

In main analyses, we excluded individuals with unknown parental occupational position (N = 27 [1.6%] in YFS and N = 15 [2%] in SKIPOGH), father not working for health reasons (N = 1 in SKIPOGH) and missing CRP (N = 1 in SKIPOGH) for a total of 1,623 YFS and 706 SKIPOGH participants (mediation analysis). Parental occupational position (or parental education) was modelled as ordered variable, hypothesizing a dose–response relationship^33^. CRP was modelled as a continuous outcome and transformed via the natural logarithm to symmetrize its distribution. Finally, parental occupational position, DNA methylation and transcriptomic data were available in 1367 YFS and 661 SKIPOGH participants (Bayesian network scoring analysis).

### Gene selection

We conceptualized a multi-gene contribution to circulating CRP levels based on the established knowledge that CRP production in the liver is triggered by multiple factors released by leukocytes (see Supplementary Information for a discussion about the choice of not selecting the *CRP* gene only). We selected genes based on two strategies independently of the study populations. First, we selected genes based on a transcriptome-wide causal inference analysis via 2-sample Mendelian Randomization (2sMR) aiming at establishing leukocytes transcripts (exposure) driving levels of CRP in blood (outcome)^17^. 2sMR is potentially robust with respect to unmeasured confounders and exploits publicly available summary statistics of large-scale GWAS. In our study, we used summary statistics of the associations between about 10 M genetic instruments (Single Nucleotide Polymorphisms, SNPs) and gene transcription in leukocytes or CRP levels in blood obtained via GWAS conducted by the eQTLGen consortium^20^ (N = 31,684) and the CHARGE consortium^21^ (N = 148,164), respectively (see Supplementary Information).

Critical to 2sMR are assumptions about validity of genetic variants as instrumental variables^35^. To satisfy the assumption that chosen variants are predictive of the exposure, we selected only eQTLs (*P* value < 1.9 × 10^–5^ corresponding to a false discovery rate < 0.05)^20^ and—to reduce bias due to reverse causation—discarded those with a strong association to CRP (*P* value < 1.9 × 10^–5^). Remaining assumptions were addressed by using *cis* eQTLs (likely direct effect of SNP on nearby gene transcription) and applying three complementary 2sMR methods^35^. First, the SNP-to-gene transcription and SNP-to-CRP associations were combined in a random effect meta-analysis using inverse variance weighting. This method takes into account the potential heterogeneity of multiple variants, but provides biased estimates when the net pleiotropic effect is different from zero or variant’s association with the exposure is dependent in magnitude from its pleiotropic effects (violation of so-called InSIDE—Instrument Strength Independent of Direct Effect—assumption)^36^. Second, we conducted MR Egger regression, which provides a consistent estimate even if the average pleiotropic effect is different from zero, but requires the InSIDE assumption. Third, we conducted penalized weighted median regression analyses, which can provide a consistent estimate for the true causal effect when up to half of the weight in the MR analysis comes from genetic variants violating all assumptions. Given the orthogonality in assumptions, if the results from these three MR methods are broadly consistent, then our causal inference is strengthened.

All 2sMR methods were run with a minimum of 10 instruments (see Supplementary Information). We estimated *F*-statistics to assess instruments strengths (*F*-stat > 10 is suggestive of strong instruments). We computed the *q* value^37^ from the *P* values estimated via the three 2sMR methods and declared a gene transcription to be driving CRP levels when *q* ≤ 0.05 in all three 2sMR methods.

Alternatively, we defined our set of genes as those having been used in previous research to assess the conserved transcriptional response to adversity (CTRA) transcriptome profile^18^. This set of CTRA indicator transcripts is composed by 53 genes characterized by up/down-regulation depending on whether their immune function is inflammation- or antiviral/antibody-related, respectively^10^. We chose this gene set as it has been associated to both early life and adult socioeconomic conditions^10,18^, and has been shown to be involved in the pathogenesis of multiple chronic diseases^38^. Additionally, those CTRA indicator genes are subject to physiological regulation by glucocorticoid signalling and pro-inflammatory stimuli^39^.

### Functional enrichment

We run gene ontology (GO) and pathways enrichment analysis of the list of genes selected via 2sMR with g:Profiler^40^. The list of genes tested via 2sMR was submitted as the background gene list and multiple testing correction was performed with the g:SCS algorithm that takes into account the structure of functionally annotated gene sets^40^. GOs or pathways were declared as significantly enriched when g:SCS adjusted *P* values were < 0.05.

### Mediation approach

We adopted a counterfactual mediation framework based on natural effect models^41^ to estimate the portion of the effect of early life socioeconomic conditions on adulthood inflammation transmitted simultaneously or *en-bloc* by transcription levels of multiple genes in leukocytes. Importantly, the identification of these joint indirect effects is not dependent on the true causal order of the mediators^42^. This property is useful in our case since the interrelations between the transcription of different genes cannot be determined unequivocally. The magnitude of total and joint indirect effects corresponded to exponentiated parameters of natural effects models and is interpretable as percentage of change in the geometric mean of CRP. The proportion mediated (PM) by a bloc of genes was derived by the ratio of the joint indirect and total effect parameters. See Supplementary Information for detailed information on interpretation and estimation of natural effect models’ parameters.

We applied principal component analysis (PCA)^43^ to the measured transcription levels of the selected genes for estimating gene transcription. In our mediation model, using principal components as gene transcription instead of measured transcription levels has the advantage of increasing the reliability of joint indirect effect estimates since it allows to both reduce the number of mediators and the impact of measurement errors in the mediators^42^. Indeed, PCA is an established dimensionality reduction method allowing the data to be described using a small number of uncorrelated variables (the principal components, PCs) while retaining as much information (variance in the original data) as possible. Furthermore, given the ordering of PCs according to decreasing values of explained variance, PCA is also a noise reduction method when retaining only the principal components with highest explained variance^43^.

In main analyses, we selected the number of principal components (PCs) in order to explain about 50% of transcription variance. Confidence intervals (95%) of total and joint indirect effects were estimated through percentiles from 5,000 bootstrap draws (with replacement).

### Cis DNA methylation

For each of the genes selected either via 2sMR or the CTRA model, we considered DNA methylation sites (CpGs) within 1,500 bp (or 5,000 bp in a sensitivity analysis) of the annotated gene location. These *cis* CpGs should ensure that their potential effect on transcription is direct and not through other genes. Furthermore, we removed cross-reactive CpGs and CpGs with known SNPs in probes having MAF > 0.01 to avoid potentially spurious or genetically driven signals.

### Bayesian network model selection

We applied Bayesian network scoring^19,24^ to select the most likely causal pathway between *cis* DNA methylation and gene transcription that are affected by early life socioeconomic conditions. For each CpG we evaluated the DAG structures represented in Figure S5. For each DAG, a posterior probability was estimated through the Bayesian Gaussian score and uniform priors across the eleven DAGs (*bnlearn* R package^19^). Finally, to alleviate the potential brittleness of inference from a large number of causal structures, we assessed posterior probabilities of 5 groups of DAGs, by specifying a partition of the DAGs^44^. Namely, group A encapsulates models where paternal occupational position does not influence DNA methylation nor transcription levels; group B encapsulates models where paternal occupational position causes changes in DNA methylation and transcription levels, and DNA methylation also causes changes in transcription; group C encapsulates models where paternal occupational position drives transcription levels, but methylation and transcription levels are not related; group D encapsulates models where paternal occupational position drives transcription levels that in turn influences methylation levels; group E encapsulates models where paternal occupational position influences DNA methylation but not transcription levels (see Figure S5). To select the group of DAGs with substantial evidence, we computed a Bayes factor (BF) from the two highest posterior probabilities and choose the group with the natural logarithm of BF larger than 1. In other words, a group of DAGs is selected only if its posterior probability is at least roughly three times larger than any other group’s posterior probability^45^.

### Sensitivity analyses

We investigated the stability of analyses upon removing participants with CRP > 10 mg/L, which may reflect short term immune responses. We computed indirect effects when summarizing transcription levels with 30% and 60% of explained variance by principal components to assess whether the effect sizes reported in main analyses underestimate the amount of mediation. In SKIPOGH, we run mediation analysis with a binary outcome (CRP ≥ 3 mg/L) as the so defined heightened inflammation was independent of the left censoring and consequently of the imputation procedure (see Table 1).

To assess the potential path-specific effect of parental occupational position on adult CRP levels not mediated by individual occupational position in adulthood (see Supplementary Information), we applied a sequential mediation approach^46^. Specifically, we estimated the indirect effect when only adulthood occupational position was included as a mediator in the first step, followed by an estimation of the indirect effect when both adulthood occupational position and transcription levels of genes in leukocytes were included as mediators. Given the posited causal structure in Fig. S6, a substantive additional indirect effect provided by both mediators is suggestive of a pathway going from paternal occupational position to CRP via transcription levels of genes in leukocytes not passing through adulthood occupational position.

To assess the potential path-specific effect of parental occupational position on adult CRP levels not mediated by leukocyte composition in blood, we applied a sequential mediation approach^46^. Specifically, we estimated the indirect effect when only leukocyte composition in blood was included as a mediator in the first step, followed by an estimation of the indirect effect when both leukocyte composition in blood and transcription levels of genes in leukocytes were included as mediators. Given the posited causal structure in Fig. S7, a substantive additional indirect effect provided by both mediators is suggestive of a pathway going from paternal occupational position to CRP via transcription levels of genes in leukocytes not passing through leukocyte composition in blood, or in other words of an effect mediated truly by levels of transcription and not proportion of leukocyte types.

We ran mediation and Bayesian scoring analyses when parental education was used as the exposure to evaluate the robustness of findings upon the choice of a different proxy for early life socioeconomic conditions. To test the ability of Bayesian network model selection to identify plausible dependency structures from the data, we repeated the model selection via Bayesian scoring when adding a group of negative control DAGs, namely models where uncoupled DNA methylation and gene transcription drive (both or either one) paternal occupational position. We ran Bayesian scoring when leukocyte composition was included as a potential confounder of the relationship between methylation and transcription levels, by adjusting for the proportion of each cell type when estimating residuals (see Supplementary Information). Finally, we ran Bayesian scoring on *cis* CpGs within 5,000 bp of the annotated gene location to evaluate the sensitivity of findings about the role of local DNA methylation in the regulation of gene transcription with respect to the choice of the threshold used to select *cis* CpGs.

## Supplementary Information

Supplementary Information.

Supplementary Table S1.

Supplementary Table S2.

## Data Availability

Summary statistics for eQTLs were downloaded from https://eqtlgen.org/. Summary statistics for CRP are available upon request to the CHARGE consortium. YFS and SKIPOGH individual data are available from the PIs of the study upon request.
