# Supplementary material for: Gene regulation contributes to explain the impact of early life socioeconomic disadvantage on adult inflammatory levels in two cohort studies
Source: Sci Rep. 2021 Feb 4;11:3100. doi: 10.1038/s41598-021-82714-2 (PMC7862626; doi:10.1038/s41598-021-82714-2)
Supplement: Supplementary file 1 — Supplementary Information. [file 41598_2021_82714_MOESM1_ESM.docx]

**Gene regulation contributes to explain the impact of early life socioeconomic disadvantage on adult inflammatory levels in two cohort studies**

Cristian Carmeli^1,2*^, Zoltán Kutalik^2,3^, Pashupati P. Mishra^4^, Eleonora Porcu^2,3,5^, Cyrille Delpierre^6^, Olivier Delaneau^7^, Michelle Kelly-Irving^6^, Murielle Bochud^2^, Nasser A. Dhayat^8^, Belen Ponte^9^, Menno Pruijm^10^, Georg Ehret^11^, Mika Kähönen^12^, Terho Lehtimäki^4^, Olli T. Raitakari^13,14,15^, Paolo Vineis^16^, Mika Kivimäki^17^, Marc Chadeau-Hyam^16^, Emmanouil Dermitzakis^18^, Nicolas Vuilleumier^19^, Silvia Stringhini^2,20^

^1^ Population Health Laboratory, University of Fribourg, Fribourg, Switzerland

^2^ Center for Primary Care and Public Health (Unisanté), University of Lausanne, Lausanne, Switzerland

^3^ Swiss Institute of Bioinformatics, Lausanne, Switzerland

^4^ Department of Clinical Chemistry, Fimlab Laboratories, and Finnish Cardiovascular Research Center, Faculty of Medicine and Health Technology, Tampere University, Tampere 33520, Finland

^5^ Center for Integrative Genomics, University of Lausanne, Lausanne, Switzerland

^6^ INSERM, UMR1027, Toulouse, France, and Université Toulouse III Paul-Sabatier, Toulouse, France

^7^ Department Computational Biology, University of Lausanne, Lausanne, Switzerland

^8^ Department of Nephrology and Hypertension, Inselspital, Bern University Hospital, University of Bern, Switzerland

^9^ Service of Nephrology, Geneva University Hospitals, Geneva, Switzerland

^10^ Service of Nephrology, Lausanne University Hospital and University of Lausanne, Lausanne, Switzerland

^11^ Department of Cardiology, Geneva University Hospital, Geneva, Switzerland

^12^ Department of Clinical Physiology, Fimlab Laboratories, and Finnish Cardiovascular Research Center, Faculty of Medicine and Health Technology, Tampere University, Tampere 33521, Finland

^13^ Centre for Population Health Research, University of Turku and Turku University Hospital, Turku, Finland

^14^ Research Centre of Applied and Preventive Cardiovascular Medicine, University of Turku, Turku, Finland

^15^ Department of Clinical Physiology and Nuclear Medicine, Turku University Hospital, Turku, Finland

^16^ MRC Centre for Environment and Health, School of Public Health, Imperial College London, W21PG UK

^17^ Department of Epidemiology and Public Health, University College London, London, UK

^18^ Department of Genetic Medicine and Development, University of Geneva, Geneva, Switzerland

^19^ Division of Laboratory Medicine, Geneva University Hospital, Geneva, Switzerland

^20^ Unit of Population Epidemiology, Division of Primary Care, Geneva University Hospitals, Geneva, Switzerland

*Correspondence:

Dr. Cristian Carmeli, PhD,

Route des Arsenaux 41, 1700 Fribourg, Switzerland.

E-mail: cristian.carmeli@unifr.ch

Tel.: +41 26 300 94 75

**SUPPLEMENTARY INFORMATION**

*Early life socioeconomic conditions*

Parental occupational position defined as the highest household occupation was used as an indicator of socioeconomic conditions in early life (main analysis). In YFS, the occupational position (manual, lower-grade non-manual and higher-grade non-manual, farmer) of the head of the household was assessed in 1980 (study’s baseline). In SKIPOGH, participants reported the profession of their father when they were children at the follow-up survey. Paternal occupational position is a commonly used indicator of socioeconomic conditions in early life^1^ and is closely related to parental occupational position in the Swiss context until the early 1980s. Indeed, according to census data^2^, in 1970 the percentage of non-working women living in a household with children (0-13 years) was 73%, and the percentage of those working full time was 8%. In 1980, the percentage of non-working women living in a household with children (0-13 years) was 67%, and the percentage of those working full time was 12%. SKIPOGH participants were born on average in 1963, with 75% of participants being born by 1978.

Parental education defined as tertiles of a score computed from paternal and maternal education was also used as an exposure (sensitivity analysis). In YFS, years of education of both the mother and the father were assessed in 1980, and they were summed up to estimate a score of parental education. In SKIPOGH, participants reported the highest attained levels of education of their mother/father into 9 levels (from no diploma to university degree). The score was computed by summing up father/mother levels so ranging between 2 (lowest) and 18 (highest).

*Adulthood occupational position*

In YFS, the participants’ occupational position (manual, lower-grade non-manual and higher-grade non-manual, farmers) was assessed in 2001 follow-up. In SKIPOGH, participants reported their profession at baseline and follow-up. We used responses from the follow-up wave and whenever participants declared to be unemployed, retired or the response was missing we used data from the baseline. Occupational position was categorized into high, intermediate, or low according to the same ESEC schema used for the parental occupational position (see Main Text).

*Systemic inflammatory levels*

In YFS, serum CRP was assessed using an automated analyzer (Olympus AU400; Tokyo, Japan) and a highly sensitive turbidimetric immunoassay kit (‘CRP‐UL’‐assay; Wako Chemicals, Neuss, Germany). Detection limit of the assay was 0.06 mg/L. The inter-assay coefficient of variation was 3.3% at 1.5 mg/L and 2.7% at 2.5 mg/L.

In SKIPOGH, CRP levels in plasma were measured at follow-up through standard immunoturbidimetry with different detection limits (1 mg/L or 2 mg/L or 3 mg/L) depending on the center or diagnostic machine used (Cobas 8000 Roche Diagnostics, Modular Roche Diagnostics, Beckmann). Inter-assay coefficients of variation were 7.5% at 10 mg/L for N=317 (Beckmann), 3.3% at 1.5 mg/L for N=372 (Modular Roche), and 4.0% at 0.9 mg/L for N=344 (Cobas 8000 Roche). Since ~60% of SKIPOGH participants had CRP values below the detection limits, we imputed left censored values via censored maximum likelihood multiple imputation^3^. This method allows to accomodate different values of detection limits in multiple batches and in simulation studies performed well even in heavy censoring scenarios as in our study^3^. We run 30 imputed data sets with a model of CRP including paternal occupational position, BMI, age, sex, and center as predictors (*lodi* R package^3^).

*DNA methylation and transcriptome*

Laboratory procedures. In YFS, 2.5 mL of whole blood was collected into PaXgene tubes (PreAnalytix, Hombrechtikon, Switzerland). Each tube was inverted 8–10 times and then stored at room temperature for at least 2 h. PaXgene tubes were frozen and stored for <1 year at −80 °C. After thawing, tubes were stored at RT 2–12 h as instructed by the manufacturer. RNA was then isolated with the PAXgene Blood RNA Kit (Qiagen) with the DNase Set according to the manufacturer’s instructions. A QiaCube isolation robot was used. The transcription levels were analyzed with an Illumina HumanHT-12 version 4 Expression BeadChip (Illumina Inc.) containing 47,231 expression and 770 control probes. In brief, 200 ng of RNA was reverse-transcribed into cDNA and biotin-UTP-labeled using the Illumina TotalPrep RNA Amplification Kit (Ambion); 1500 ng of cDNA was then hybridized to the Illumina HumanHT-12 v4 Expression BeadChip. The BeadChips were scanned with the Illumina HiScan system. Raw Illumina probe data were exported from Beadstudio and analyzed in R 3.1.2 using the Bioconductor 3.0 packages. The transcriptomic data were processed using a nonparametric background correction, followed by quantile normalization with control and expression probes, and a log2 transformation. The transcriptomic analysis was successful for 1,650 individuals.

In YFS, leukocytes’ DNA was obtained from EDTA-blood samples using a Wizard® Genomic DNA Purification Kit (Promega Corporation, Madison, WI, USA) according to the manufacturer’s instructions. In 179 YFS participants genome-wide DNA methylation was interrogated through the Illumina Infinium HumanMethylation450 BeadChip, while in 1,369 individuals DNA methylation was measured through the Illumina Infinium MethylationEPIC BeadChip (1,548 individuals in total). Raw data were processed according to the CPACOR pipeline^4^. Leukocyte composition was estimated via the Houseman method^5^, leading to proportions of T helper cells, cytotoxic T cells, natural killer cells, beta cells, monocytes, and granulocytes.

In SKIPOGH, whole blood was collected in PAXgene tubes (PreAnalytiX, Humbrechtikon, Switzerland), handled according to the manufacturer's instructions and stored at -80 °C. RNA was extracted using the Qiagen PAXgene Blood RNA kit (Qiagen, Venlo, Netherlands) with quality assessment and quantification by Qbit (ThermoFisher, Waltham, Massachusetts). RNA was sequenced via Illumina HiSeq 2000. Total reads per individual were on average 26,854,567 (with a standard deviation of 5,731,567). Mapping of reads was performed with GEM algorithm^6^ and resulted in an average successful mapping of 73.2% (with a standard deviation of 5.6%) of the total reads. Gene counts quantification was performed via QTLtools^7^. Counts were transformed through the *voom*^8^ procedure and normalized via trimmed mean of *M*-values. The transcriptomic analysis was successful for 723 individuals.

DNA was extracted using standard methods on a bead-based KingFisher Duo robot extraction system (ThermoFisher, Waltham, Massachusetts). DNA quality assessment and quantification was performed using a Nanodrop system (ThermoFisher, Waltham, Massachusetts). For bisulfite conversion, the protocol started with ~1.2ug of DNA extracted. For the PCR step: alternative incubation conditions were performed when using the Illumina Infinium® Methylation Assay (Appendix page 6 of bisulfite conversion protocol pdf). The final elution was done with 8uL of M-Elution Buffer. In 250 SKIPOGH participants genome-wide DNA methylation was interrogated through the Illumina Infinium HumanMethylation450 BeadChip, while in 451 individuals DNA methylation was measured through the Illumina Infinium MethylationEPIC BeadChip (701 individuals in total). Raw data were processed according to the CPACOR pipeline^4^. Leukocyte composition was estimated via the Houseman method^5^. To compare the estimated proportions of leukocyte types with those measured experimentally, we used the measured proportions of granulocytes, lymphocytes, and monocytes in 378 SKIPOGH participants. We evaluated the agreement between the measured and estimated proportions using the Spearman correlation coefficient. As reported in Figure S8, the correlation values ranged from 0.56 to 0.84, supporting a good agreement between measured and estimated proportions.

Statistical analyses. For mediation analyses, in SKIPOGH gene transcription’s residuals were obtained by removing the effects of RNA integrity number and mean GC. For Bayesian network scoring analyses, we estimated gene transcription’s residuals by removing the effects of age, sex, centre of blood collection and family membership (random intercept, only in SKIPOGH). In analyses involving DNA methylation data, we transformed the proportion of methylated cells into *M*-values and then estimated residuals after adjustment for chip type (Illumina Infinium HumanMethylation450 or MethylationEPIC BeadChips), first thirty principal components of control probes, age, sex, centre of blood collection and family membership (random intercept, only in SKIPOGH).

*Summary statistics databases*

The eQTLGen database includes summary statistics for 153,437,930 associations of 10,823,015 SNPs nearby (<1Mbp) 16,989 genes. Associations between SNPs and gene transcription levels were computed and pooled across 37 cohorts for a total of 31,684 adult individuals. Gene transcription levels were profiled by arrays or RNA sequencing (20.3% of individuals). SNPs with a gene-level *P* value<1.9*10^-5^ (equivalent to permutation false discovery rate <0.05) are considered as *cis* expression quantitative trait loci (*cis* eQTLs)^9^. The CHARGE database includes summary statistics for 10,019,203 SNPs from pooled associations of 49 1000G imputed-genotype GWAS (N=148,164 adults). Serum CRP was measured in mg/L and natural-log transformed^10^.

*2-sample Mendelian Randomization methods*

For each gene, available *cis* eQTLs were clumped by imposing a linkage disequilibrium *r*^2^<0.25. The correlation between instruments was taken into account by running inverse-variance weighted methods^11^. All 2sMR methods were run with a minimum of 10 instruments. We estimated *F*-statistics to assess instruments strengths (*F*-stat>10 is suggestive of strong instruments).

We computed the *q* value^12^ from the *P* values estimated via the three 2sMR methods. We declared a gene transcription to be driving CRP levels (at any period before adulthood) when *q*≤0.05 in all three 2sMR methods. Mendelian randomization was run via the *TwoSampleMR*^13^ and *Mendelian Randomization*^11^ R packages. *Q* values were estimated via the *qvalue* R package.

*Counterfactual mediation approach*

We adopted a counterfactual mediation framework to disentangle direct and indirect effects of the exposure(s) on the outcome, via multiple mediators. Contrary to traditional path analysis based on parametric structural equation modelling, mediation analysis based on counterfactuals relies on formal causal arguments and it allows path tracing even with binary outcomes, whereas the traditional path analysis rely on stringent parametric constraints^14^. In this study, we fitted marginal natural effect models, a class of marginal structural models for parametrizing and estimating so-called natural direct and indirect effects^15^.

Let nested counterfactual Y(a, M(a*)) denote the CRP value that would have been observed if exposure were set (potentially counter to fact) to low parental occupational position (denoted by a) and mediators M to the value they would have taken if exposure were set (potentially counter to fact) to high parental occupational position (denoted by a*). Denoting as C the set of confounders and hypothesizing no product terms, we posited the following natural effect model

$$log\left[ E\left\{ Y\left( a, M\left( a^{*} \right) \right)|C \right\} \right]=\beta_{0}+\beta_{1}a+\beta_{2}a^{*}+\beta_{3}C$$

From it, we can simultaneously estimate the natural direct effect (% of change in geometric mean of Y) as

$(exp\{\beta_{1}(a-a^{*})\}$ – 1)*100

and the natural joint indirect effect (% of change in geometric mean of Y) as

$(exp\{\beta_{2}(a-a^{*})\}$ – 1)*100.

The sum $(exp\{\beta_{1}\left( a-a^{*} \right)+ \beta_{2}\left( a-a^{*} \right)\}$ – 1)*100 measures the total effect and the ratio $\frac{\beta_{2}}{(\beta_{1}+\beta_{2})}$ measures the proportion mediated.

Under certain identifying conditions (no unmeasured confounders of exposure-outcome, exposure-mediators, mediators-outcome, and mediator-mediator induced by exposure relations), the effect of exposure on outcome mediated simultaneously by all mediators (so-called joint indirect effect) can be estimated^16^. Importantly, identification of joint indirect effects is not dependent on the true causal order of the mediators^17^. In other words, joint indirect effects are robust to potential misspecifications of the causal order among the examined mediators. This property is useful in our case since the interrelations between the transcription levels of different genes cannot be determined unequivocally.

In counterfactual terms, the effect mediated by transcription of all genes taken together or *en-bloc* (see Figure 2A) represents the effect on CRP values when altering the levels of the mediators from levels observed if exposed to high parental occupational position to levels that would have been observed at low parental occupational position while otherwise remaining exposed to low parental occupational position.

The aforementioned joint effects correspond to parameters of natural effects models (see above) and were estimated via an imputation model of the nested counterfactuals^18^. This was performed in three steps by: i) fitting linear regression models for log-transformed CRP with parental occupational position, the mediators and confounders as explanatory variables; ii) imputing the counterfactual log-CRP for each combination of unobserved (counterfactual) exposures and observed mediators’ levels; iii) fitting a linear natural effect model for imputed log-CRP. In all cases, we specified models for the outcome imputation to reflect the structure of the natural effects models^18^. Under the assumption of an outcome imputation model correctly specified and congenial with the natural effect model^18^, this estimation approach is appealing because it is parsimonious in that it requires specifying only one model even in the presence of multiple mediators. Furthermore, in empirical simulations, it provided adequate estimates compared to inverse probability and doubly robust estimators^18^.

Mediation analyses were performed using *medflex* R package^20^.

*CRP distribution in the two populations*

As reported in Table 1, CRP levels in SKIPOGH were distributed differently than in YFS, as participants in the upper quintile had CRP≥3 mg/L in SKIPOGH while had CRP>2.2 mg/L in YFS. The reasons underlying the observed difference in the distribution of CRP between the two cohorts are potentially multiple. As the difference in the age distribution is substantial between the two cohorts and it is very well known that CRP values increase with age, the inter-cohort difference could well be explained by the fact that SKIPOGH participants are older (53y) than YFS participants (42y). Another potential explanation could be that in YFS there are fewer participants with high SES (18%) than in SKIPOGH (25%), as it is well established that low SES individuals have higher CRP values than high SES individuals. Furthermore, there is some evidence that men may have higher CRP values than women^20^. Finally, we cannot rule out whether differences in the assays are also contributing to those CRP differences. In a study on about 800 angiographically studied patients, high sensitivity CRP had a median value lower than standard CRP values^21^.

*CRP gene selection*

We have conceptualized a multi-gene and not a single-gene contribution to circulating CRP levels. As the expression of the *CRP* gene in the liver drives circulating CRP levels, we could have included the *CRP* gene in our analyses. However, our transcription data were measured from blood, not from liver. Additionally, the 2sMR analysis could not be performed on the *CRP* gene. In fact, there were no valid genetic instruments in the eQTLgen database, as none of the measured eQTLs of the *CRP* gene were strong instruments with a P value < 1.9*10^-5^. Given these two notions, we did not investigate the *CRP* gene as we could not reliably conclude whether the expression of the *CRP* gene in leukocytes drives circulating CRP levels or vice versa.

**REFERENCES**

1. Galobardes, B., Lynch, J. & Smith, G. D. Measuring socioeconomic position in health research. *Br Med Bull* **81-82**, 21-37, doi:10.1093/bmb/ldm001 (2007).
2. Swiss Federal Statistical Office [Internet]. Activité professionnelle, tâches domestiques et familiales. Available from: <https://www.bfs.admin.ch/bfs/fr/home/statistiques/population/familles.html>
3. Boss, J. *et al.* Estimating Outcome-Exposure Associations when Exposure Biomarker Detection Limits vary Across Batches. *Epidemiology* **30**, 746-755, doi:10.1097/EDE.0000000000001052 (2019).
4. Lehne, B. *et al.* A coherent approach for analysis of the Illumina Methylation450 BeadChip improves data quality and performance in epigenome-wide association studies. *Genome Biol* **16**, 37 (2015).
5. Houseman, E. A. *et al.* DNA methylation arrays as surrogate measures of cell mixture distribution. *BMC Bioinformatics* **13**, 86 (2012).
6. Marco-Sola, S., Sammeth, M., Guigó, R. & Ribeca, P. The GEM mapper: fast, accurate and versatile alignment by filtration. *Nat Methods* **9**, 1185-1188 (2012).
7. Delaneau, O. *et al.* A complete tool set for molecular QTL discovery and analysis. *Nat Comm* **8**, 15452 (2017).
8. Ritchie, M. E. *et al.* Limma powers differential expression analyses for RNA-sequencing and microarray studies. *Nucleic Acids Res* **43**, e47 (2015).
9. Võsa, U. *et al.* Unraveling the polygenic architecture of complex traits using blood eQTL metaanalysis. *bioRxiv*, 447367, doi:10.1101/447367 (2018).
10. Ligthart, S. *et al.* Genome Analyses of >200,000 Individuals Identify 58 Loci for Chronic Inflammation and Highlight Pathways that Link Inflammation and Complex Disorders. *Am J Hum Genet* **103**, 691-706, doi:10.1016/j.ajhg.2018.09.009 (2018).
11. Burgess, S., Nutterworth, A. S. & Thompson, S. G. Mendelian randomization analysis with multiple genetic variants using summarized data. *Genet Epidemiol* **37**, 658-665 (2013).
12. Storey, J. D. The positive false discovery rate: a Bayesian interpretation and the q-value. *Annals of Statistics* **31**, 2013-2035 (2003).
13. Hemani, G. *et al.* The MR-Base platform supports systematic causal inference across the human phenome. *Elife* **7**, e34408 (2018).
14. MacKinnon, D. P. & Dwyer, J. H. Estimating mediated effects in prevention studies. *Eval Rev* **17**, 144-158 (1993).
15. Lange, T. S., Vansteelandt, S. & Bekaert, M. A simple approach for estimating natural direct and indirect effects. *Am J Epidemiol* **176**,190-195 (2012).
16. Steen, J., Loeys, T., Moerkerke, B. & Vansteelandt, S. Flexible mediation analysis with multiple mediators. *Am J Epidemiol* **186**, 184-193 (2017).
17. VanderWeele, T. J. & Vansteelandt, S. Mediation analysis with multiple mediators. *Epidemiol Methods* **2**, 95-115 (2014).
18. Vansteelandt, S., Bekaert, M. & Lange, T. Imputation strategies for the estimation of natural direct and indirect effects. *Epidemiol Methods* **1**, 131-158 (2012).
19. Steen, J., Loeys, T., Moerkerke, B. & Vansteelandt, S. medflex: An R Package for Flexible Mediation Analysis using Natural Effect Models. *Journal of Statistical Software* **76**, 1-46 (2017).
20. Tang, Y. *et al*. The baseline levels and risk factors for high-sensitivie C-reactive protein in Chinese healthy population. *Immun Ageing* **15**, 21 (2018).
21. Clarke, J.L. *et al*. Comparison of differing C-reactive protein assay methods and their impact on cardiovascular risk assessment. *Am J Cardiol* **95**, 155-158 (2005).


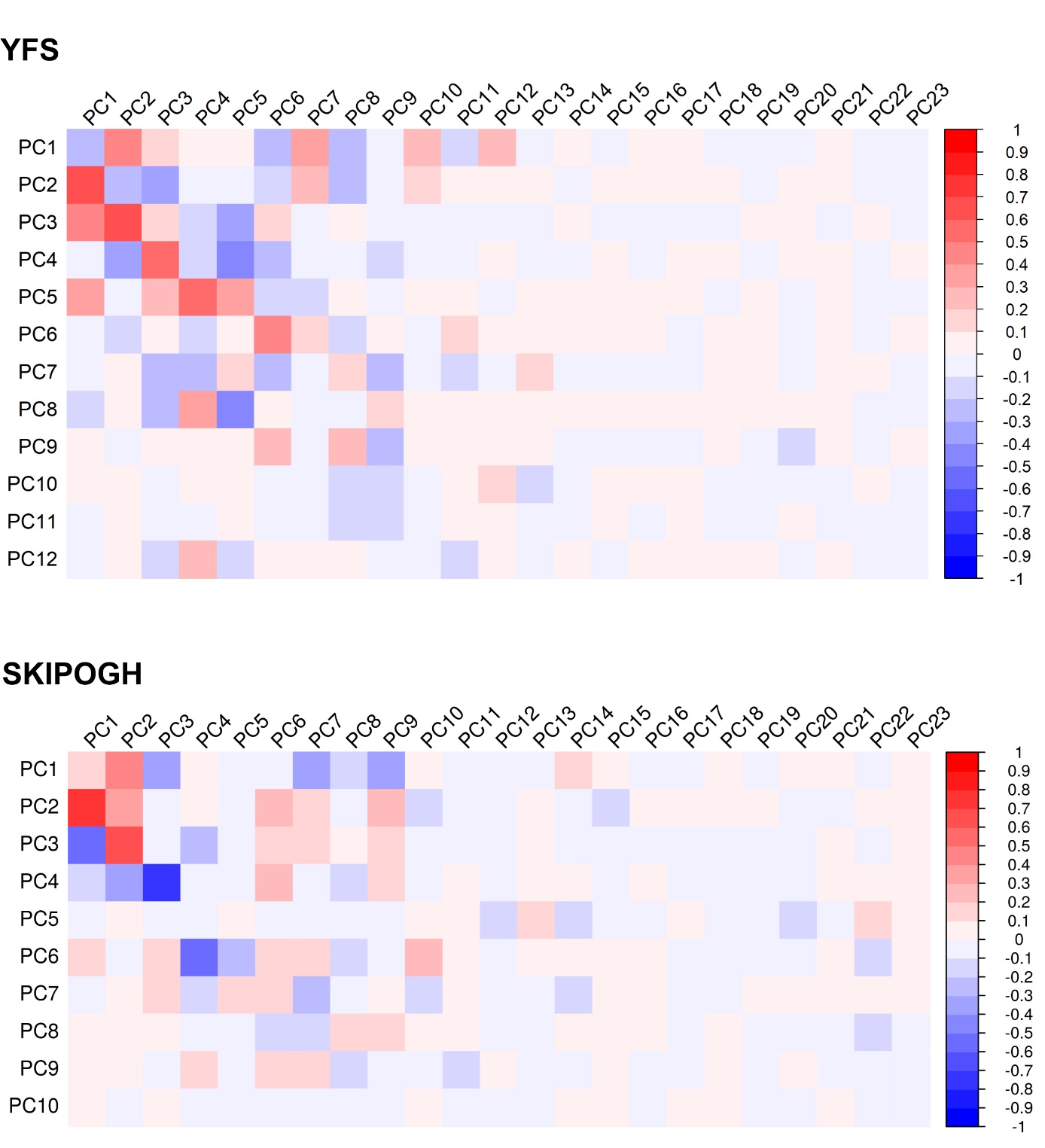


**Figure S1**. Heatmap of Pearson’s correlation between principal components of transcription levels of 2sMR genes (columns) and of CTRA indicator genes (rows) in CRYFS (top) and SKIPOGH (bottom) participants. Warm colours are for positive correlation values, while cold colours are for negative values. The PCs summarize about 50% of the transcription levels variance in the 2sMR (23 PCs in both CRYFS and SKIPOGH) and CTRA (12 PCs in CRYFS and 10 PCs in SKIPOGH) sets, respectively. The PCs are ordered according to decreasing amounts of explained variance.


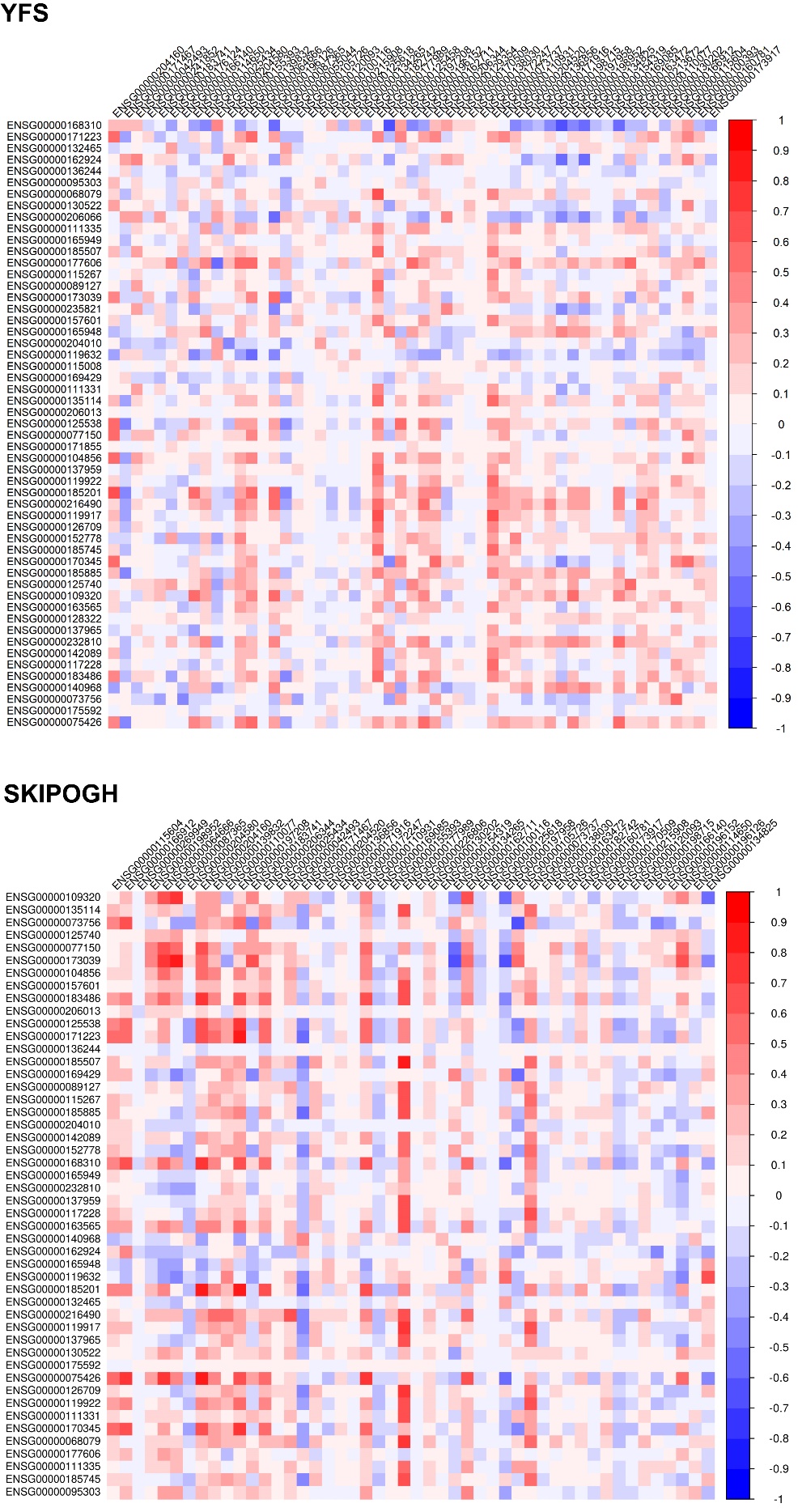


**Figure S2**. Heatmap of Pearson’s correlation between transcription levels of 2sMR genes (columns) and of CTRA indicator genes (rows) in YFS (top) and SKIPOGH (bottom) participants. Warm colours are for positive correlation values, while cold colours are for negative values. Genes are ordered according to their similarity in transcription levels based on hierarchical clustering run separately in both sets. Genes are reported according to their Ensembl identifier. In YFS, four 2sMR genes are not reported as they were not measured. In SKIPOGH, five CTRA genes are not reported as they had an average value of counts per million reads smaller than 0.25.


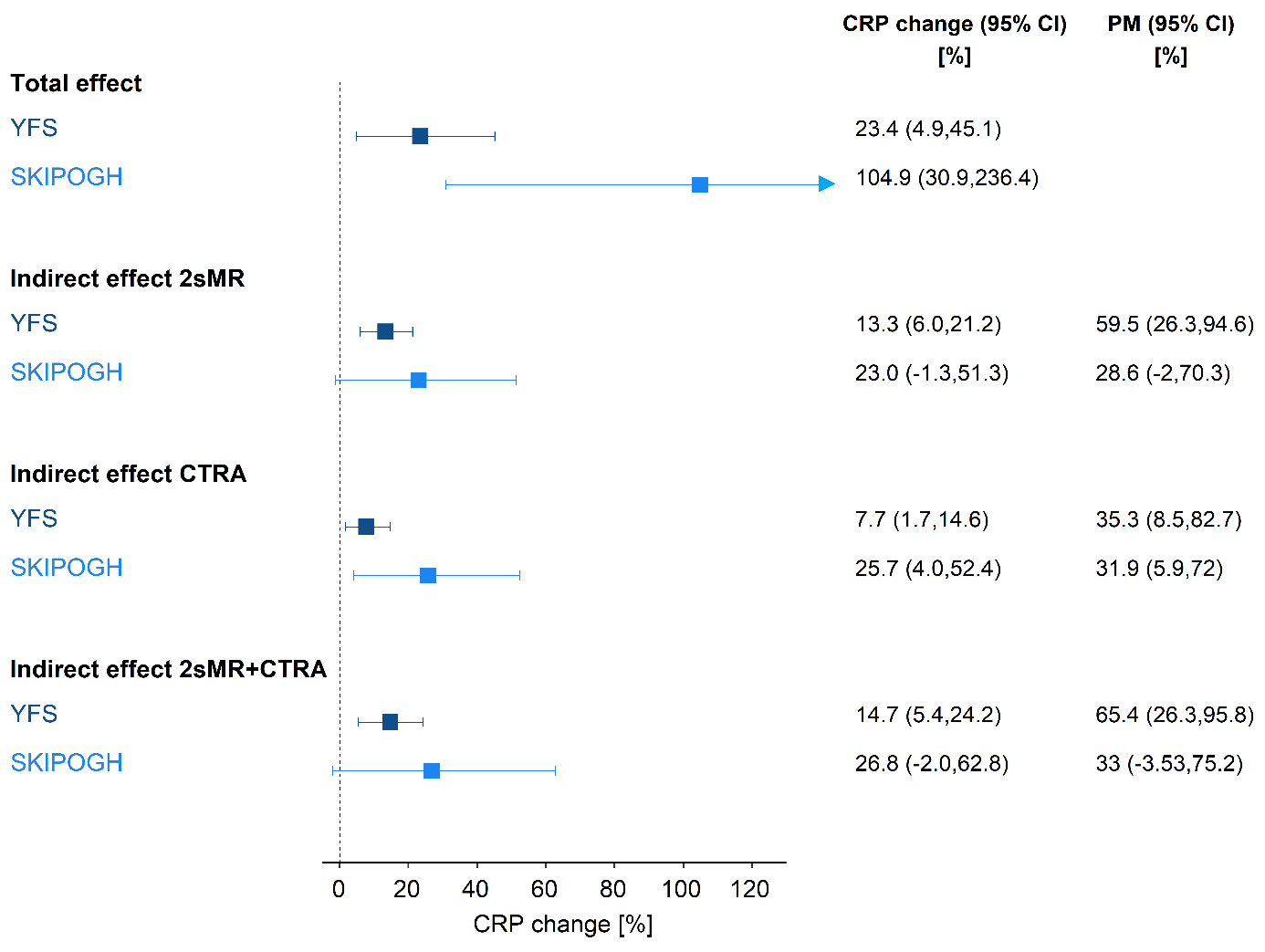


**Figure S3**. Size of effects and proportion mediated (PM) estimated by mediation analysis in each population when participants with CRP>10 mg/L were excluded (N=34 in YFS and N=11 in SKIPOGH). CRP change (%) and 95% confidence intervals (CI) are reported for the total effect of low (vs high) parental occupational position on CRP levels in adulthood, and for indirect effects through transcription levels of selected genes *en-bloc*. Joint indirect effects via 2sMR, CTRA or 2sMR+CTRA genes were estimated by summarizing transcription levels with principal components corresponding to about 50% of the transcription variance in each genes’ set.


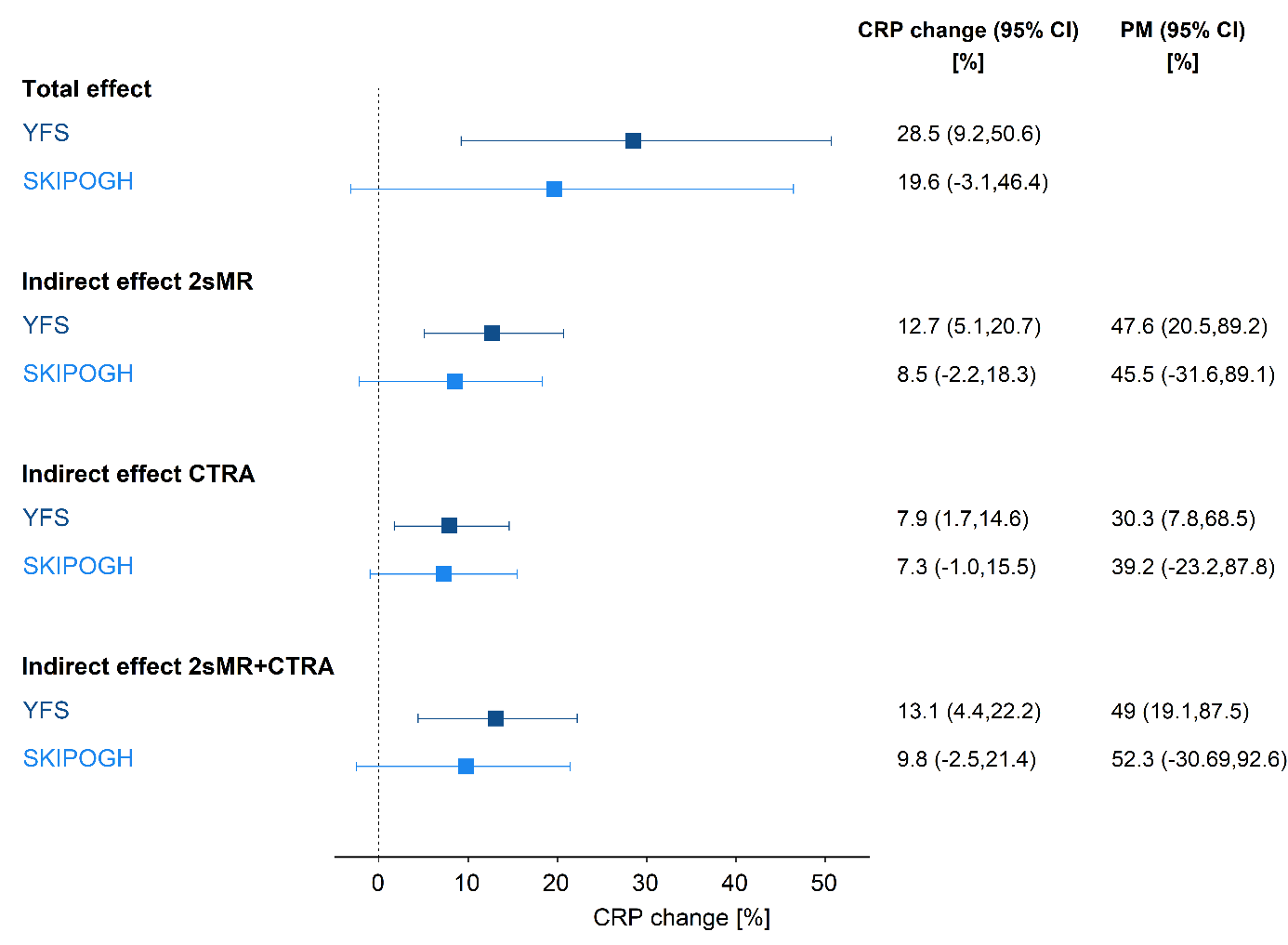


**Figure S4**. Size of effects and proportion mediated (PM) estimated by mediation analysis in each population when parental education was the exposure (N=1,433 in YFS and N=721 in SKIPOGH). CRP change (%) and 95% confidence intervals (CI) are reported for the total effect of low (vs high) parental education on CRP levels in adulthood, and for indirect effects through transcription levels of selected genes *en-bloc*. Joint indirect effects via 2sMR, CTRA or 2sMR+CTRA genes were estimated by summarizing transcription levels with principal components corresponding to about 50% of the transcription variance in each genes’ set.


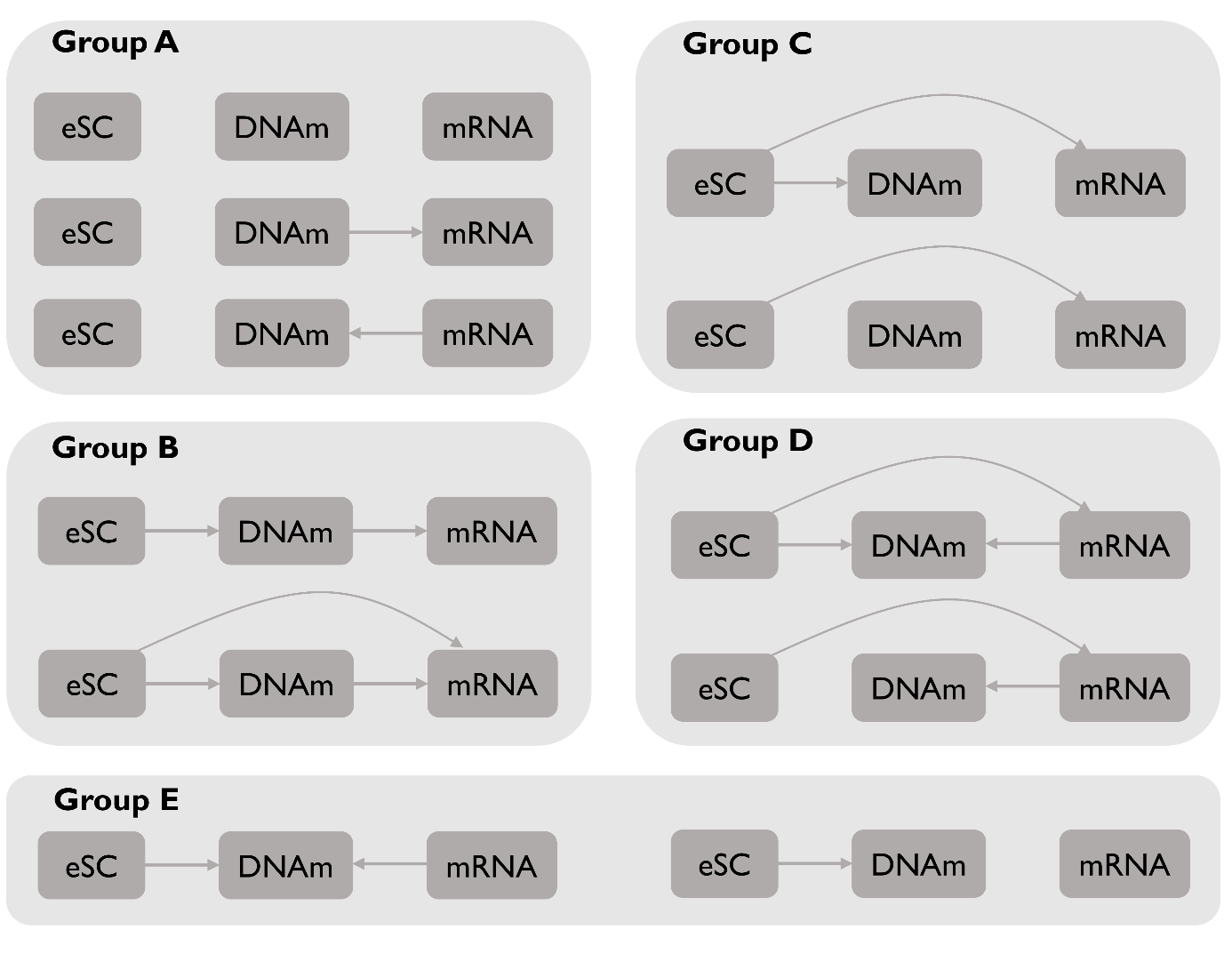


**Figure S5**. Competing groups of causal structures (DAGs) to investigate the relationships between early life socioeconomic conditions (eSC), methylation at DNA sites (DNAm) and gene transcription (mRNA) in leukocytes in adulthood. For the sake of simplicity potential confounders (age, sex, centre of blood collection) are not drawn.


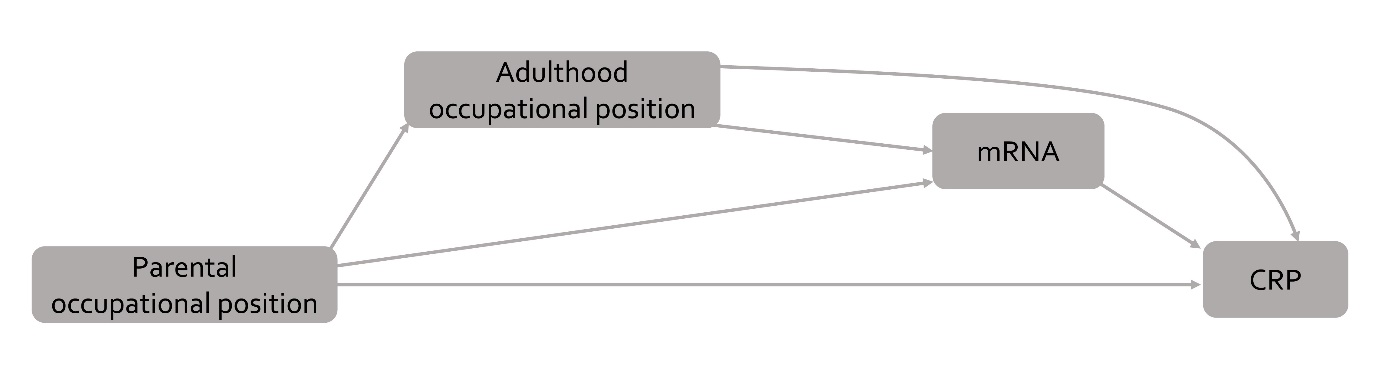


**Figure S6.** Causal structure (DAG) to investigate path-specific effects of early life socioeconomic conditions (parental occupational position) on inflammatory levels in adulthood (CRP). Parental occupational position represents the exposure, CRP represents the outcome, and adulthood occupational position and transcription of genes in leukocytes (mRNA) the investigated intermediate mechanisms. For the sake of simplicity, we do not draw confounders (age, sex, centre of blood collection).

**
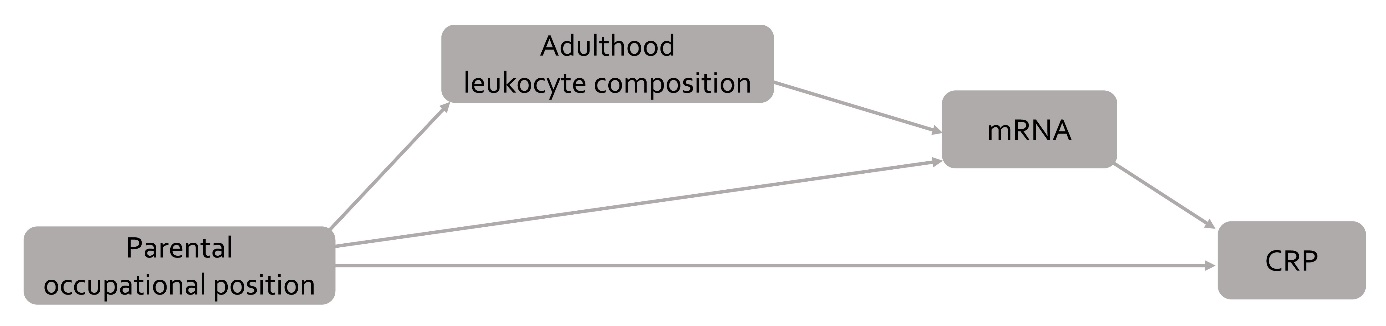
**

**Figure S7.** Causal structure (DAG) to investigate path-specific effects of early life socioeconomic conditions (parental occupational position) on inflammatory levels in adulthood (CRP). Parental occupational position represents the exposure, CRP represents the outcome, and adulthood leukocyte composition and transcription of genes in leukocytes (mRNA) the investigated intermediate mechanisms. For the sake of simplicity, we do not draw confounders (age, sex, centre of blood collection).

**
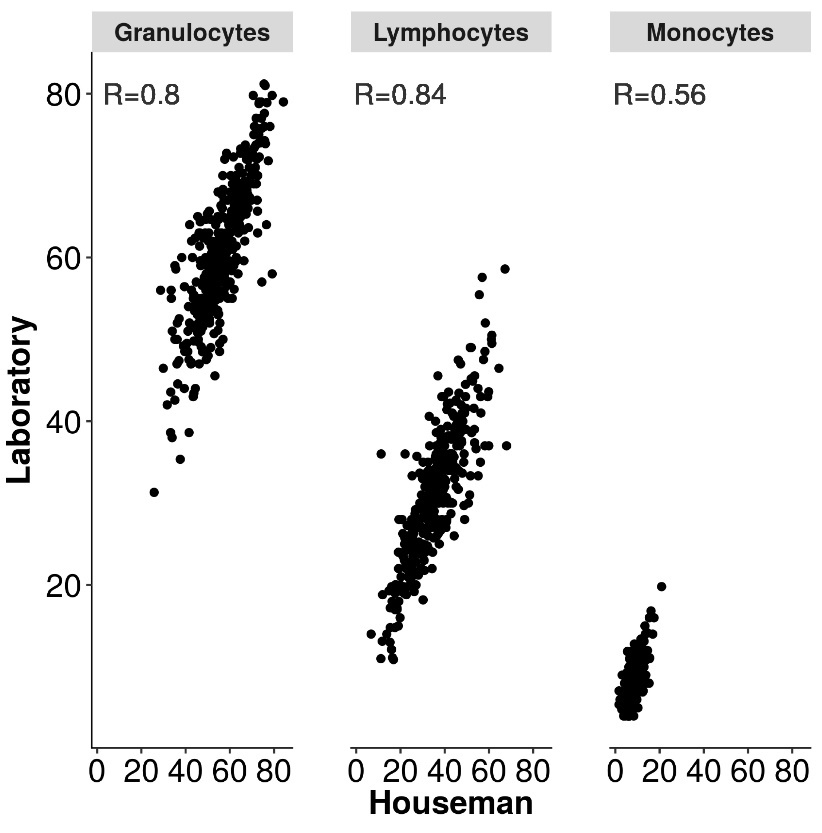
**

**Figure S8.** Scatterplot of measured and estimated leukocyte composition in 378 SKIPOGH participants. Three cell types were measured, namely granulocytes, lymphocytes and monocytes (from left to right). On the y-axis is the proportion (percentage) measured in the laboratory, while on the x-axis is the proportion (percentage) estimated from DNA-methylation data through the Houseman deconvolution method. The correlation value displayed on the left top corner represents the Spearman correlation.

**Table S1.** Annotation (HGNC symbol and Ensembl name, genomic location in GRCh37) of 2sMR and CTRA genes.

**Table S2.** Annotation (identifier, genomic position in GRCh37, and Ensembl identifier of nearby gene) and winning model (5 models, see Figure S5) of 4,076 CpGs selected for the Bayesian network scoring analysis.

**Table S3**. Joint indirect effects via 2sMR-implicated genes and CTRA indicator genes (% of CRP change) when transcription levels are summarized via different numbers of principal components corresponding to about 30%, 40%, 50% or 60% of transcription variance.

| **Explained transcriptional variance** | **YFS** | **SKIPOGH** |
| --- | --- | --- |
| 30% | 12.0% (3.9%,20.8%) | 10.4% (0.2%,19.9%) |
| 40% | 14.0% (5.1%,24.0%) | 11.1% (-0.7%,23.1%) |
| 50% | 17.2% (7.1%,28.5%) | 13.9% (0.0%,27.2%) |
| 60% | 17.3% (6.7%,29.1%) | 16.0% (1.0%,32.2%) |

**Table S4**. Size of effects (odds ratio OR) and proportion mediated (PM) estimated by mediation analysis in SKIPOGH when binarizing CRP values (≥3 mg/L for heightened inflammation). OR and 95% confidence intervals (CI) are reported for the total effect of low (vs high) parental occupational position on heightened inflammation in adulthood, and for indirect effects through transcription levels of 2sMR+CTRA genes *en-bloc*. Transcription levels were summarized with principal components corresponding to about 50% of the transcription variance in the genes’ set.

| **Effects** | **OR (95% CI)** |
| --- | --- |
| Total effect | 2.22 (1.33,3.74) |
| Indirect effect 2sMR+CTRA | 1.47 (1.06,1.95); PM = 48.1% (8.9%,89.0%) |

**Table S5**. Size of effects estimated by mediation analysis in each population when parental occupational position was the exposure, adulthood occupational position and transcription levels of 2sMR+CTRA genes in leukocytes were the mediators (N=1,245 in YFS and N=683 in SKIPOGH). CRP change (%) and 95% confidence intervals (CI) are reported for the total effect of low (vs high) parental occupational position on CRP levels in adulthood, and for indirect effects through adulthood occupational position only and adulthood occupational position and transcription levels of 2sMR+CTRA genes. Transcription levels of 2sMR+CTRA genes were summarized with principal components corresponding to about 50% of the transcription variance in the genes’ set.

| **Effect** | **YFS** | **SKIPOGH** |
| --- | --- | --- |
| Total effect | 28.1% (4.3%,57.4%) | 65.7% (32.5%,106.9%) |
| Indirect effect through occupational position in adulthood | 7.5% (1.9%,13.9%) | 3.9% (-1.2%,10.6%) |
| Indirect effect through adulthood occupational position and transcription levels of 2sMR+CTRA genes in leukocytes | 26.5% (4.3%,57.4%) | 15.5% (0.2%,30.4%) |

**Table S6**. Size of effects estimated by mediation analysis in each population when parental occupational position was the exposure, adulthood leukocyte composition and transcription levels of 2sMR+CTRA genes in leukocytes were the mediators (N=1,367 in YFS and N=660 in SKIPOGH). CRP change (%) and 95% confidence intervals (CI) are reported for the total effect of low (vs high) parental occupational position on CRP levels in adulthood, and for indirect effects through leukocyte composition only, and through both leukocyte composition and transcription levels of 2sMR+CTRA genes. Leukocyte composition was estimated through the Houseman method providing six cell proportions (see DNA methylation and transcriptome paragraphs). The six cell proportions were analysed as a mediator *en-bloc*. Transcription of 2sMR+CTRA genes were summarized with principal components corresponding to about 50% of the transcription variance in the genes’ set.

| **Effect** | **YFS** | **SKIPOGH** |
| --- | --- | --- |
| Total effect | 22.8% (1.7%,47.0%) | 52.1% (26.0%,85.2%) |
| Indirect effect through leukocyte composition in adulthood | -2.4% (-6.0%,1.0%) | -0.8% (-6.7%,2.8%) |
| Indirect effect through adulthood leukocyte composition and transcription levels of 2sMR+CTRA genes in leukocytes | 18.0% (6.7%,29.3%) | 11.6% (-1.6%,24.6%) |

**Table S7.** Cardinality of the most likely group of model structures across the five tested competing groups (see Figure S5) for a total of 4,076 CpG sites when parental education was used as exposure in up to N=1,211 YFS and N=677 SKIPOGH participants. The most likely group of causal structures is selected when its posterior probability is at least roughly three times larger than any other group’s posterior probability. None corresponds to CpGs for which none of the five tested groups reached winning evidence or models could not be estimated as the transcription level was not available in a few genes (37 and 33 CpGs in YFS and SKIPOGH, respectively).

| **Causal structures** |  | **N of CpGs** | |
| --- | --- | --- | --- |
|  |  | **YFS** | **SKIPOGH** |
| A | Parental education does not influence methylation nor transcription levels | 3,599 | 3,527 |
| B | Parental education drives changes in methylation and transcription levels; methylation also causes changes in transcription | 0 | 0 |
| C | Parental education drives gene transcription, but methylation and transcription are not related | 47 | 4 |
| D | Parental education drives transcription that in turn influences methylation levels | 1 | 0 |
| E | Parental education influences methylation but not transcription levels | 4 | 28 |
| None |  | 425 | 517 |
| Total |  | 4,076 | 4,076 |

**Table S8.** Cardinality of the most likely group of model structures across the five tested competing groups (see Figure S5) for a total of 4,076 CpG sites when parental occupational position was used as exposure in up to N=1,367 YFS and N=661 SKIPOGH participants, and leukocyte composition is added as a confounder of the relationship between methylation and transcription levels. The most likely group of causal structures is selected when its posterior probability is at least roughly three times larger than any other group’s posterior probability. None corresponds to CpGs for which none of the five tested groups reached winning evidence or models could not be estimated as the transcription level was not available in a few genes (37 and 33 CpGs in YFS and SKIPOGH, respectively).

| **Causal structures** |  | **N of CpGs** | |
| --- | --- | --- | --- |
|  |  | **YFS** | **SKIPOGH** |
| A | Parental occupational position does not influence methylation nor transcription levels | 3,524 | 3,130 |
| B | Parental occupational position drives changes in methylation and transcription levels; methylation also causes changes in transcription | 0 | 0 |
| C | Parental occupational position drives gene transcription, but methylation and transcription are not related | 38 | 42 |
| D | Parental occupational position drives transcription that in turn influences methylation levels | 1 | 2 |
| E | Parental occupational position influences methylation but not transcription levels | 9 | 35 |
| None |  | 504 | 867 |
| Total |  | 4,076 | 4,076 |

**Table S9.** Cardinality of the most likely group of model structures across the five tested competing groups (see Figure S5) for a total of 4,771 CpG sites, selected as being within 5,000 bp of the annotated gene location. Parental occupational position was used as exposure in up to N=1,367 YFS and N=661 SKIPOGH participants, and leukocyte composition is added as a confounder of the relationship between methylation and transcription levels. The most likely group of causal structures is selected when its posterior probability is at least roughly three times larger than any other group’s posterior probability. None corresponds to CpGs for which none of the five tested groups reached winning evidence or models could not be estimated as the transcription level was not available in a few genes.

| **Causal structures** |  | **N of CpGs** | |
| --- | --- | --- | --- |
|  |  | **YFS** | **SKIPOGH** |
| A | Parental occupational position does not influence methylation nor transcription levels | 4,224 | 3,842 |
| B | Parental occupational position drives changes in methylation and transcription levels; methylation also causes changes in transcription | 0 | 2 |
| C | Parental occupational position drives gene transcription, but methylation and transcription are not related | 33 | 45 |
| D | Parental occupational position drives transcription that in turn influences methylation levels | 12 | 24 |
| E | Parental occupational position influences methylation but not transcription levels | 11 | 28 |
| None |  | 483 | 830 |
| Total |  | 4,771 | 4,771 |
